# A Novel Cyanobacterium *Synechococcus elongatus* PCC 11802 has Distinct Genomic and Metabolomic Characteristics Compared to its Neighbor PCC 11801

**DOI:** 10.1038/s41598-019-57051-0

**Published:** 2020-01-13

**Authors:** Damini Jaiswal, Annesha Sengupta, Shinjinee Sengupta, Swati Madhu, Himadri B. Pakrasi, Pramod P. Wangikar

**Affiliations:** 1 Department of Chemical Engineering, Indian Institute of Technology Bombay, Powai, Mumbai, 400076 India; 2 DBT-PAN IIT Centre for Bioenergy, Indian Institute of Technology Bombay, Powai, Mumbai, 400076 India; 3 Department of Biology, Washington University, St. Louis, MO 63130 USA; 4 Department of Energy, Environmental and Chemical Engineering, Washington University, St. Louis, MO 63130 USA; 5 Wadhwani Research Centre for Bioengineering, Indian Institute of Technology Bombay, Powai, Mumbai, 400076 India

**Keywords:** Metabolomics, Metabolic engineering, Comparative genomics, Physiology, Sugar phosphates

## Abstract

Cyanobacteria, a group of photosynthetic prokaryotes, are attractive hosts for biotechnological applications. It is envisaged that future biorefineries will deploy engineered cyanobacteria for the conversion of carbon dioxide to useful chemicals via light-driven, endergonic reactions. Fast-growing, genetically amenable, and stress-tolerant cyanobacteria are desirable as chassis for such applications. The recently reported strains such as *Synechococcus elongatus* UTEX 2973 and PCC 11801 hold promise, but additional strains may be needed for the ongoing efforts of metabolic engineering. Here, we report a novel, fast-growing, and naturally transformable cyanobacterium, *S. elongatus* PCC 11802, that shares 97% genome identity with its closest neighbor *S. elongatus* PCC 11801. The new isolate has a doubling time of 2.8 h at 1% CO_2_, 1000 µmole photons.m^−2^.s^−1^ and grows faster under high CO_2_ and temperature compared to PCC 11801 thus making it an attractive host for outdoor cultivations and eventual applications in the biorefinery. Furthermore, *S. elongatus* PCC 11802 shows higher levels of key intermediate metabolites suggesting that this strain might be better suited for achieving high metabolic flux in engineered pathways. Importantly, metabolite profiles suggest that the key enzymes of the Calvin cycle are not repressed under elevated CO_2_ in the new isolate, unlike its closest neighbor.

## Introduction

Cyanobacteria are a group of prokaryotes that are capable of carrying out oxygenic photosynthesis. These microorganisms have gained attention in the field of biotechnology due to their efficient photoautotrophy, genetic amenability, and the potential for direct conversion of carbon dioxide (CO_2_) to useful products. Engineered cyanobacteria have been reported for the production of a wide variety of chemicals, albeit at titers that are well below those needed for commercial production^1–9^. Majority of these studies have employed model strains such as *Synechocystis* sp. PCC 6803, *Synechococcus elongatus* PCC 7942, and *Synechococcus* sp. PCC 7002 (henceforth referred to as PCC 6803, PCC 7942, and PCC 7002, respectively, for brevity). Substantial information is now available on transcriptome, proteome, metabolome, and synthetic biology tools of these model cyanobacteria^10–15^. Thus, these strains and other closely related cyanobacteria would be favorable choices as hosts for chemical production. Further, it is believed that fast-growing strains that afford more efficient photoautotrophy will be better suited as hosts for metabolic engineering^16–18^. Moreover, organisms that are tolerant to high temperatures, light, and CO_2_ levels are desirable as industrial strains considering the eventual outdoor utilization in biorefineries. Thus, it would be of interest to expand the repertoire of cyanobacterial strains by adding strains that fulfill these criteria. Phylogenetic proximity to the widely studied model strains would be an added advantage.

Recently, fast-growing cyanobacterial strains *Synechococcus elongatus* UTEX 2973 and *S. elongatus* PCC 11801 (henceforth UTEX 2973 and PCC 11801, respectively) have been reported^19,20^. Their growth rates are higher than model cyanobacterial strains like PCC 6803, PCC 7942, and PCC 7002^19,20^. Detailed growth, biochemical characterization, and systems biology data is now available for UTEX 2973 that was reported in 2015^18,21,22^. Likewise, the physiological and proteome changes in PCC 11801 under elevated CO_2_ conditions have been studied^14,19^. However, more comprehensive transcriptomic, proteomics, and metabolomics research is needed to characterize the strain PCC 11801 in detail. Furthermore, it would be of interest to develop a library of well-characterized synthetic parts like promoters, ribosomal binding sites (RBS), and terminators that can be used in the new hosts according to the requirement for a given product and in a particular environment^10,23,24^. For example, a gene for toxic metabolic intermediate can be expressed under a weak or tuneable promoter so that the metabolite does not accumulate inside the cell. Unlike the heterotrophic counterparts like *E. coli* and yeast, there are only a limited number of fast-growing cyanobacterial strains such as UTEX 2973, PCC 11801, and PCC 7002. Therefore, it is envisaged that some new cyanobacteria need to be isolated and characterized for metabolic engineering applications, and the present work is a step in that direction^25^.

Studies involving metabolomics and metabolic flux analysis are helpful in identifying the bottlenecks in the metabolic pathways that can be potential targets for strain engineering^9,12,26,27^. For example, a comparison of relative metabolic pool sizes across different conditions may provide valuable biological insights on the potential rate-limiting steps in the metabolic network under a particular condition^28–30^. Depending on the inherent metabolism of a cyanobacterial strain, the yield of desired product may differ among different strains. However, there are no reports of a head-on comparison of yields for a particular product by expressing a given pathway across different strains^11^. Likewise, there are limited reports on comparative metabolite profiling of different cyanobacterial strains under conditions that are expected to result in higher productivity^29,31^.

In this study, we report *Synechococcus elongatus* PCC 11802 (henceforth referred to as PCC 11802), which is a close relative of PCC 11801, also isolated from Powai Lake, Mumbai, India (19.1273°N, 72.9048°E). Detailed physiological, genomic, and metabolic characterization of PCC 11802 is presented here. Unlike its closest neighbor PCC 11801 that has its least doubling time of 2.3 h under ambient CO_2_ conditions^19^, PCC 11802 has a least doubling time of 2.8 h at 38 °C, 1% CO_2_ and a light intensity of 1000 µmole photons.m^−2^.s^−1^. The two strains thus have different CO_2_ specificities when it comes to their optimal growth conditions. PCC 11802 is also naturally transformable similar to PCC 11801. This study reports detailed physiological and genomic characterization of PCC 11802 with a focus on differences between PCC 11802 and PCC 11801. Metabolite profiling of PCC 11802 and PCC 11801 under elevated CO_2_ conditions uncovers the differences in their metabolic capabilities for carbon assimilation. This study adds one more cyanobacterial strain in the phylogenetic neighborhood of the widely studies PCC 7942 and a potential candidate for metabolic engineering.

## Results and Discussion

### Identification and genome analysis of PCC 11802

**Figure 1:**
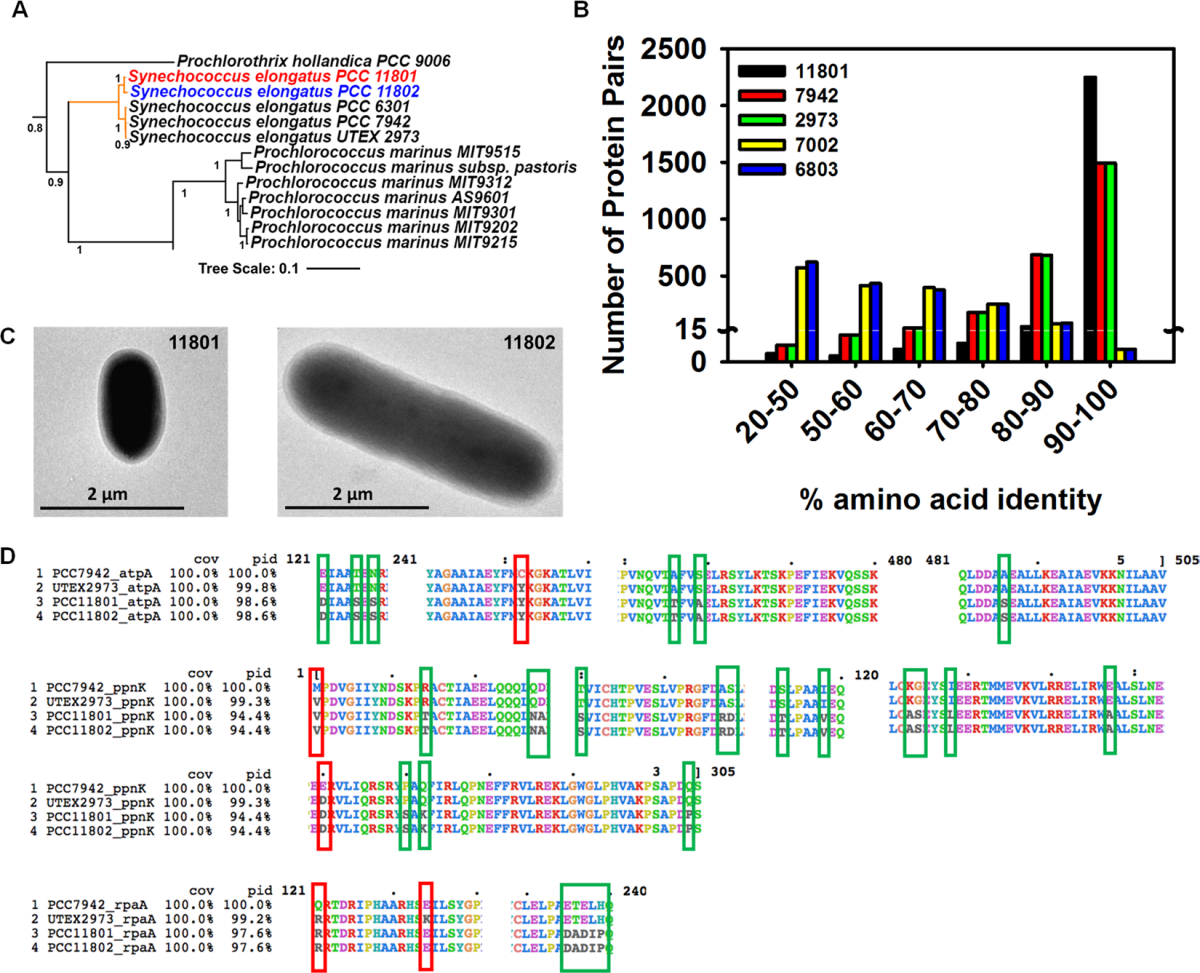
Identification, phylogenetic assignment, and genome comparison of PCC 11802. (**A**) A phylogenetic tree created using concatenated protein sequences of 29 constitutive genes showing the completely sequenced organisms in the phylogenetic neighborhood of PCC 11802. A complete tree with 128 cyanobacteria is shown in the supplementary information (Fig. S2). (**B**) A histogram depicting the level of amino acid identity between shared genes between PCC 11802 genome and the cyanobacterial strains PCC 11801, PCC 7942, UTEX 2973, PCC 7002 and PCC 6803. Only bidirectional best hits were included in the statistics. (**C**) Cell size comparison of exponentially growing cultures of PCC 11802 and PCC 11801 and (**D**) Alignment of sequences of proteins, AtpA, PpnK and RpaA showing the amino acid substitutions responsible for fast-growth phenotype and other single amino acid polymorphisms (SAPs) observed in PCC 11802 and 11801 with respect to PCC 7942 and UTEX 2973.

**Figure 2:**
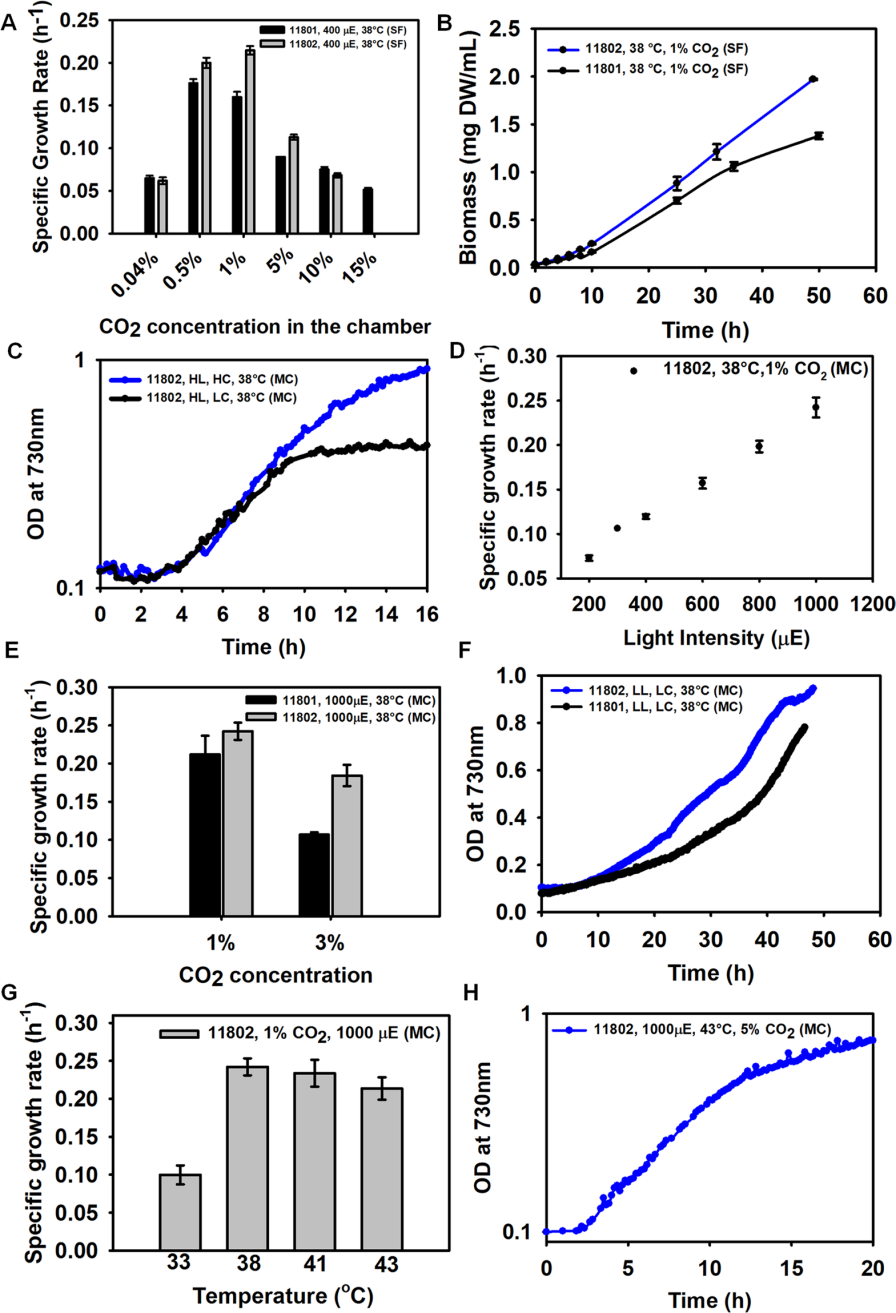
Growth characterization of PCC 11802 and comparison with PCC 11801. (**A**) The growth rates of PCC 11802 and 11801 in shake flasks (SF) under 400 µmole photons.m^−2^.s^−1^, 38 °C and varied CO_2_ levels in the chamber, (**B**) biomass accumulation of PCC 11802 compared to that of PCC 11801 at 1% CO_2_ in SF, (**C**) growth of PCC 11802 at high light (HL, 1000 µmole photons.m^−2^.s^−1^), high CO_2_ (HC, 1% CO_2_) compared with growth at high light (HL) and low CO_2_ (LC, 0.04% CO_2_) at 38 °C in MC, (**D**) dependence of specific growth rate of PCC 11802 on the light intensity at 38 °C and 1% CO_2_ in MC, (**E**) specific growth rates of PCC 11802 compared to PCC 11801 at high light (1000 µmole photons.m^−2^.s^−1^) and high CO_2_ concentrations (1% and 3%) at 38 °C in MC, (**F**) comparison of growth of PCC 11802 to PCC 11801 under low light (LL, 200 µmole photons.m^−2^.s^−1^) and low CO_2_ (LC, 0.04% CO_2_) at 38 °C, (**G**) specific growth rates of PCC 11802 at varying temperatures at 1000 µmole photons.m^−2^.s^−1^ and 1% CO_2_ in MC, and (**H**) growth profile of PCC 11802 at 1000 µmole photons.m^−2^.s^−1^, 43 °C and 5% CO_2_ in MC.

PCC 11802 was isolated along with PCC 11801 and six other strains from Powai Lake, Mumbai, India (19.1273°N, 72.9048°E). All the eight strains showed growth rates higher than the closest model strain PCC 7942. The detailed characterization of PCC 11801 has been reported earlier^19^. Here, we report the characteristics of PCC 11802 and its comparison to its close neighbor PCC 11801 and the model strain PCC 7942. The phylogenetic analysis using 16S rRNA and concatenated sequences of a set of 29 house-keeping proteins^32,33^ revealed that PCC 11802 belonged to *Synechococcus elongatus* clade (Figs. 1A, S1 and S2). The strain was deposited with Pasteur Culture Collection of Cyanobacteria as *Synechococcus elongatus* PCC 11802. The average length and width calculated by imaging the exponentially growing cells (OD_730_ ~ 0.5–0.6) of PCC 11802 were found to be 3.8 and 1.2 µm, respectively. The cell length of PCC 11802 is thus significantly larger than PCC 11801 that has an average length and width of 2.5 and 1.4 µm, respectively (Fig. 1C)^19^. It has been reported that the cell size of cyanobacteria can be affected by growth rate^34^. However, we believe that the difference observed in the cell sizes of PCC 11801 and 11802 is not due to differences in growth rate as the imaging was performed under ambient CO_2_ conditions at 38 °C where both the strains have comparable growth rates (Fig. 2A).

The whole-genome sequencing of PCC 11802 revealed a genome size of 2.7 Mbp. The genome of PCC 11802 was annotated using three different annotation servers, *viz*., Rapid Annotation using Subsystem Technology (RAST)^35–37^, Integrated Microbial Genomes and Microbiomes^38^, JGI (IMG) and NCBI Prokaryotic Genome Annotation Pipeline^39^. The general characteristics of the annotated genome are presented in Table 1 (refer to the Supplementary File S-1 for annotation). PCC 11802 shares ~83% and ~97% genome identity with PCC 7942 and PCC 11801, respectively (Figs. S3–S6). There are 94,540 single nucleotide polymorphisms (SNPs) in PCC 11802 compared to PCC 11801 (Supplementary File S-2). Around 48,486 of these SNPs are present in the coding regions of the genome that contribute to 19,481 amino acid changes in the proteins. However, there are 338,422 SNPs in PCC 11802 compared to PCC 7942 (Supplementary File S-3). Next, it was of interest to analyze the amino acid identity between the corresponding homologous proteins of PCC 11802 and some of the model strains (Fig. 1B). As expected, PCC 11802 shares the highest identity with proteins of PCC 11801, followed by PCC 7942 and UTEX 2973 (Fig. 1B and Table S1). On the other hand, majority of the proteins of PCC 11802 showed fairly low identity with the corresponding homologs from PCC 7002 and PCC 6803 (Fig. 1B and Table S1).

**Table 1:** Genome annotation for *Synechococcus elongatus* PCC 11802 obtained using RAST, IMG, and NCBI prokaryotic genome annotation pipeline.

	RAST	IMG	NCBI Prokaryotic Genome Annotation Pipeline
*Gene count*	2924	2919	2752
*GC content*	54.8	54.8	—
*Protein coding genes*	2883	2866	2708
*Protein with known function*	2127	2201	2446
*Protein without known function*	756	665	262
*Total RNA coding genes*	41	53	44
*tRNA genes*	39	39	39
*rRNA genes*	2	3	2
*Other RNAs*	—	11	3

It has been of significant interest to identify the genetic determinants of fast growth phenotype. To that end, Pakrasi and coworkers demonstrated that of the 53 single nucleotide polymorphisms (SNPs) between UTEX 2973 and PCC 7942, the SNPs in the genes, *atpA, ppnK*, and *rpaA* are responsible for the fast growth of UTEX 2973^21,40–42^. Of the five single amino acid polymorphisms (SAPs) present in these three genes of UTEX 2973, four SAPs were also found in PCC 11801 and PCC 11802 (Fig. 1D). In addition to these SAPs, we detected six, fifteen, and five other SAPs in the protein sequences of genes, *atpA, ppnK*, and *rpaA*, respectively, in PCC 11801 and PCC 11802 compared to UTEX 2973 and PCC 7942. The functional significance of these additional SAPs in PCC 11802 and PCC 11801 cannot be explained presently. We further analyzed the 31 conserved house-keeping proteins^32^ of PCC 11802 against the respective proteins of PCC 11801 and PCC 7942 (Figs. S7–34). The sequences of proteins, RpsJ, RpsS, and RplK do not have any mutation among these three strains. We found an average amino acid identity of ~97% between PCC 11802 and PCC 7942 for these 31 conserved proteins. On the other hand, PCC 11802 shares 99.5% amino acid identity with PCC 11801 for the 31 conserved proteins.

### Function-based genome comparison between PCC 11802 and 11801

We further investigated the differences between the two closely related strains, PCC 11802 and PCC 11801. The function-based genome comparison resulted in a set of proteins that are unique in PCC 11802 and PCC 11801 compared to each other (Table 2). We observed that the major differences between the two strains were the presence of different kinds of type II toxin-antitoxin systems (TAS). Apart from the type II TAS proteins, there were a few other proteins specific to PCC 11802 or 11801. The potential role of these proteins, as described in literature, has been summarized in Table 2. However, the functional validation of these TAS proteins in PCC 11802 will be required before an exact role can be assigned.

**Table 2:** The function-based comparison between *Synechococcus elongatus* PCC 11802 and 11801 identified using RAST tool. The genes annotated only in PCC 11802 compared to PCC 11801 genome and vice-versa are represented. The obtained results were also verified by comparing the obtained protein sequence against the whole genome sequence using the NCBI BLAST.

	Locus Tag^†^	Protein ID^†^	Putative Role	Remarks based on previous reports
**Proteins unique to PCC 11802**				
*Type III restriction-modification system methylation subunit/site-specific DNA- methyltransferase*	1823836–1821905*	peg.1952^#^	DNA methylation	May be involved in epigenetic regulation of genes through differential methylation of genes^77,78^
*Retron-type RNA-directed DNA polymerase*	EKO22_06085	QFZ92002	Reverse transcription	Uses RNA as a template as well as the primer for reverse transcription^79,80^
*Programmed cell death antitoxin MazE like*	EKO22_08965	QFZ92458	Programmed cell death/Reversible Growth Arrest	Toxin-antitoxin system (TAS) responsible for heat-induced programmed cell death in *Synechocystis* sp. PCC 6803^81^
*Programmed cell death toxin MazF like*	EKO22_08970	QFZ92459		
*RelB/StbD replicon stabilization protein (antitoxin to RelE/StbE)*	EKO22_03050	QFZ91496		Involved in the adjustment of nutrient consumption under starvation conditions^44–46^
*VapB protein (antitoxin to VapC)*	EKO22_08890	QFZ92444		
*VapC toxin protein*	EKO22_08885	QFZ92443		
*YefM protein (antitoxin to YoeB)*	EKO22_07885	QFZ92286	Survival of cells in prolonged exposure to stress	This TAS system has been shown to be involved in cell survival under prolonged exposure to antibiotics by entering a dormant state^82^
*YoeB toxin protein*	EKO22_07880	QFZ92285		
**Proteins unique to PCC 11801**				
*Putative ski2-type helicase MJ1124*	DOP62_06670	AZB72445	RNA degradation, processing, and splicing	Demonstrated to activate RNA degradation in eukaryotes^83^
*HigA protein (antitoxin to HigB)*	DOP62_07875	AZB72637	Reversible Growth Arrest	These are found to be overexpressed in *Mycobacterium tuberculosis* under different stress conditions such as heat shock, nutrient starvation, DNA damage and hypoxia^84^
*HigB toxin protein*	DOP62_07880	AZB72638		

^†^The IDs are based on annotations obtained through NCBI Prokaryotic Genome Annotation Pipeline.

^*^The coordinates of gene annotated as a single protein by RAST have been provided instead of NCBI locus tag since a translated protein sequence was not obtained using NCBI Prokaryotic Genome Annotation Pipeline.

^#^RAST protein ID is provided instead of NCBI protein ID. The protein sequence can be obtained from Supplementary File S-1 (Table S3).

The TAS has been poorly characterized in cyanobacteria with only a few reports describing their potential functions. Out of the six known TAS, type II is comparatively better characterized by well-defined genetic loci^43^. Type II TAS consists of a stable toxin protein and an antitoxin protein that loses the stability under stress conditions^44^. Under normal growth conditions, the antitoxin is synthesized in a concentration that is much higher than toxin forming a toxin-antitoxin complex, thereby inhibiting the toxicity caused by the toxin. Under stress conditions (heat, nutrient starvation and loss of plasmid), either the antitoxin production is repressed, or it becomes unstable, releasing the toxin into the cell that targets cellular machinery responsible for cell death or growth arrest^45^. Most TAS systems cause programmed cell death, growth cessation or reversible growth arrest during stressful conditions by targeting critical cellular functions like the integrity of cell membrane, cell wall synthesis, DNA replication, ribosome assembly, and translation^43,45–48^. Broadly, the function of TAS is to allow the cells to cope up with stress conditions. The presence of different type II TAS in PCC 11802 and PCC 11801 may be indicative of employment of different mechanisms for cell survival or growth arrest under a particular stress condition. These TAS can be taken up as potential targets in studies aimed at gaining a fundamental understanding of cellular behavior under unfavorable conditions. These systems should also be explored for biotechnological applications that may require growth arrest during product formation phases^49^.

### Growth characteristics of PCC 11802

The growth characterization of PCC 11802 was performed under a range of light, CO_2,_ and temperature conditions, the three major factors determining cyanobacterial growth. We monitored growth in shake flasks (SF) as well as a multi-cultivator (MC), the latter offering a wider range of light intensities, a shorter light path, and the ability to bubble gases. Among the CO_2_ levels tested for growth in SF at 38 °C, the minimum doubling time of 3.2 h was observed for PCC 11802 at 1% CO_2_ (0.04–15%) (Fig. 2A). PCC 11801, on the other hand, showed the least doubling time of 3.9 h at 0.5% CO_2_ (3.4 h for PCC 11802) under similar light and temperature conditions in SF (Fig. 2A). Note that PCC 11802 has higher growth rates compared to PCC 11801under elevated CO_2_ levels (Fig. 2A,E). Both these strains have similar growth rates at 0.04% and 10% CO_2_ in SF, while PCC 11802 was not able to grow at 15% CO_2_ (Fig. 2A). Further, PCC 11802 accumulates greater biomass compared to PCC 11801 under high CO_2_ conditions (Fig. 2B), which may be due to its higher growth rate at elevated CO_2_.

**Figure 3:**
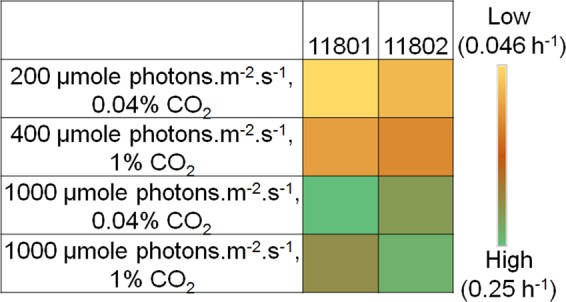
Comparison of growth rates of PCC 11802 and PCC 11801 under different combinations of light and CO_2_. The color map is created based on their growth rate values in MC at 38 °C under the respective condition without any normalization. The scale bar from low to high growth rate is represented.

It has been reported that the growth rate of PCC 11801 under ambient CO_2_ increased by ~4 fold when grown in MC with the bubbling of air compared to that in SF^19^. Therefore, we investigated whether PCC 11802 behaves similarly when grown in MC. We observed that unlike PCC 11801 that has a least doubling time of 2.3 h under ambient (0.04%) CO_2_ at 1000 µmole photons.m^−2^.s^−1^ and 41 °C_,_ PCC 11802 has its fastest growth at 1% CO_2_, 38 °C and 1000 µmole photons.m^−2^.s^−1^ with bubbling in MC (Fig. 2C,D,G). The doubling time of PCC 11802 under the optimal conditions in MC was found to be 2.8 h (Fig. 2C). The growth rates of PCC 11802 and 11801 declined when tested at 3% CO2 in MC (Fig. 2E). However, the decline in the growth of PCC 11802 was much less (~ 25%) compared to PCC 11801 (~54%). The growth of PCC 11802 under low light (200 µmole photons.m^−2^.s^−1^) and low CO_2_ (0.04%) condition was ~1.7 times faster than PCC 11801 (Fig. 2F, Table S2)^19^. PCC 11802 maintains a better growth rate (doubling time of ~3.6 h) even at a further higher level of CO_2_ (5%) at 1000 µmole photons.m^−2^.s^−1^ and 1% CO_2_ (Fig. 2H)_._ It is evident from these results that PCC 11802 has a different CO_2_ specificity and grows better at elevated CO_2_ compared to PCC 11801. It is also noticeable that the growth of PCC 11802 during the exponential phase is limited by CO_2_, whereas that of PCC 11801 is limited by light (Fig. 3). It has been demonstrated in PCC 6803 that the proteome is significantly altered in response to light compared to CO_2_^50^. Under low light and high CO_2_ conditions, the abundance of carbon assimilatory proteins reduces to compensate for expanded light-harvesting proteins^50^_._ The differential responses of PCC 11801 and PCC 11802 to the availability of light and CO_2_ might be due to differential regulatory controls, abundance, and utilization of light and carbon assimilatory proteins. We speculate that under low light conditions, PCC 11801 might allocate significant proteome fraction for light-harvesting proteins resulting in shrinking of carbon-assimilatory proteins and thus affecting the overall growth. However, a detailed comparative proteomics study on these two strains under these conditions will be required to pinpoint the differential regulatory controls.

The growth of PCC 11802 was also monitored under different temperatures at 1% CO_2_ (Fig. 2G). Both PCC 11802 and 11801 are able to tolerate a temperature of 20 °C with a doubling time of 18–19 h under ambient CO_2_ conditions (Fig. S35). We observed that PCC 11802 has similar growth at 38 °C and 41 °C (Fig. 2G, Table S2), whereas the growth of PCC 11801 increased ~16% at 41 °C^19^. PCC 11802 grows better at 43 °C with only a ~9% decline in the growth rate, whereas the growth of PCC 11801 shows a more drastic, 60% decline at 43 °C^19^. Thus, both the strains have optimal growth temperatures ranging between 38–41 °C.

### Carbon storage and energy charge under elevated CO_2_ conditions

**Figure 4:**
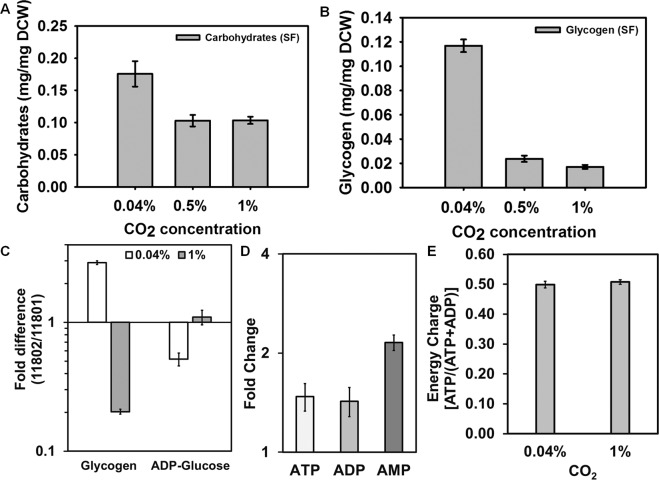
Comparison of carbon storage and energy balance between ambient and elevated CO_2_ condition. (**A**) Total carbohydrate and (**B**) glycogen content of PCC 11802 under ambient (0.04%) and elevated (0.5% and 1%) CO_2_ levels, (**C**) fold difference of glycogen and ADP-glucose (precursor for glycogen synthesis) between PCC 11802 and 11801 under ambient and 1% CO_2_, (**D**) fold change of the nucleotides ATP, ADP and AMP at 1% CO_2_ compared to ambient CO_2_ in PCC 11802 and (**E**) cellular energy charge of PCC 11802 under ambient and 1% CO_2_ condition.

The synthesis of glycogen and carbon partitioning plays an important role in energy balancing during cyanobacterial growth^51^. Consistent with our previous results in PCC 11801^19^, PCC 11802 also has lower carbohydrate and glycogen content (Fig. 4A,B) under fast growth conditions of 0.5 and 1% CO_2_ (Fig. 2A). PCC 11801 had the lowest glycogen content at 0.5% CO_2_^19^_,_ where its growth rate is maximum in SF (Fig. 2A). It is noticeable that the growth rate of PCC 11801 at 1% CO_2_ is less compared to 0.5% CO_2_ (Fig. 2A). The glycogen content of PCC 11801 at 1% CO_2_ is higher than at 0.5% CO_2_^19^_,_ signifying that the excess carbon that could not be utilized for biomass production is stored as glycogen. On the other hand, the growth of PCC 11802 is highest at 1% CO_2_ in SF (Fig. 2A) with the least glycogen content (Fig. 4B). PCC 11802 has ~3 times higher glycogen content and ~2 times less ADP-glucose (the precursor for glycogen synthesis) than PCC 11801 under ambient CO_2_ conditions (Fig. 4C). However, the glycogen content of PCC 11802 is ~5 times lower than PCC 11801 at 1% CO_2_. This suggests that PCC 11802 is more efficient than PCC 11801 in utilizing the available CO_2_ for growth rather than glycogen storage. This further strengthens our results on better growth and carbon assimilation efficiency of PCC 11802 under high CO_2_ conditions compared to PCC 11801.

PCC 11802 has elevated levels of nucleotides, ATP, ADP, and AMP at 1% CO_2_ compared to ambient CO_2_ (Fig. 4D). It is reported that the levels of these nucleotides increase before entering the exponential phase and decline steadily after the exponential phase^51^. Higher levels of these energy nucleotides at elevated CO_2_ might be indicative of better photosynthesis and ATP production. The higher growth rate under elevated CO_2_ might be facilitated by higher pools of these energy nucleotides that participate in anabolic reactions during cell growth. Despite higher levels of these energy nucleotides under high CO_2_, the energy charge at ambient and 1% CO_2_ was found to be constant (Fig. 4E). This suggests that the cells try to balance the energy charge even when the metabolite pools changes under different conditions.

### Metabolic changes under elevated CO_2_ conditions

It is of interest to identify the metabolic flux control points or the reactions that exert a high degree of control over the flux through a pathway. Among the omics tools that can be used for this purpose, metabolomics can provide useful information regarding the potential bottlenecks in a pathway^52–54^. For example, accumulation of a particular metabolite can result either from a higher rate of production or a lower rate of utilization by the downstream reactions^30^. Importantly, a rate-limiting reaction in a particular pathway can be readily identified based on the accumulation of its substrate and depletion of its product. Measurement of absolute metabolite concentrations is challenging due to matrix effects, inefficient extraction, degradation during extraction and variation in the detector sensitivity^28,55,56^. Therefore, to achieve even relative quantitation, the use of internal standards is necessary. Isotopic dilution mass spectrometry (IDMS) is a technique that can correct for artifacts in quantitation by using the peak area ratio of an analyte and its isotopic internal standard^28,55–58^.

**Figure 5:**
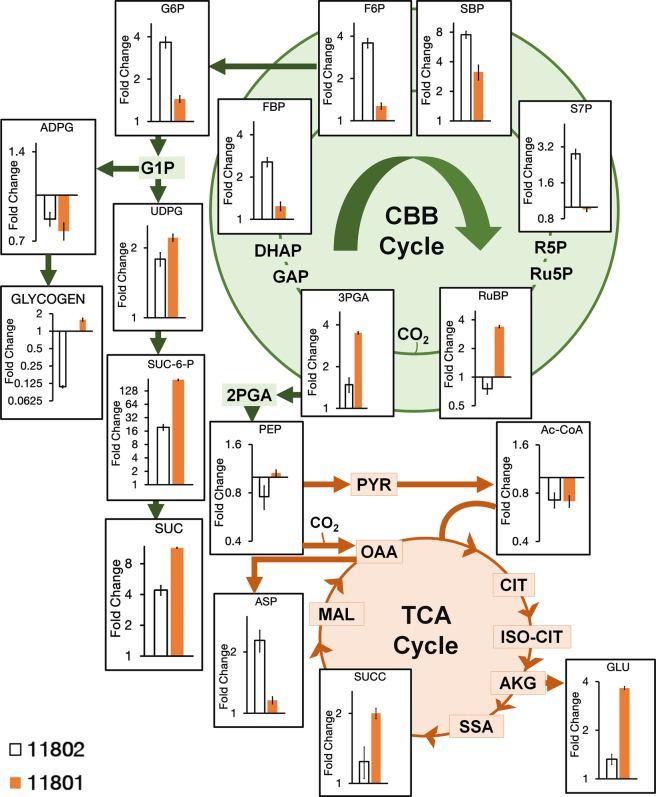
LCMS based analysis of metabolic pool sizes in PCC 11802 and 11801 under elevated CO_2_ conditions. The bar graphs represent the ratio of metabolite pools at 1% CO_2_/ambient (0.04%) CO_2_ in PCC 11802 and 11801. The metabolites represented in the figure show the difference between PCC 11802 and 11801 at a p-value < 0.01 using the t-test with the exception of phosphoenolpyruvate (PEP), acetyl coenzyme A (Ac-CoA) and ADP-glucose (ADPG) which show p-values of 0.02, 0.09 and 0.5 respectively. The relative metabolite pool sizes in each condition were estimated by extracting the metabolites as described previously and mixing with an extract of PCC 11801 which is fully labeled with isotopic ^13^C carbon. High-resolution LCMS data was collected by achieving separation of metabolites using a reverse phase, ion-pairing chromatography method. The ratio of areas of the ^12^C monoisotopic peak to the respective ^13^C monoisotopic peak was quantified for each metabolite, which served as a measure of relative pool size in a given condition. Abbreviations used for metabolites, ADPG: ADP-glucose, Ac-CoA: acetyl coenzyme A, AKG: alpha-ketoglutaric acid, ASP: aspartic acid, CIT: citric acid, DHAP: dihydroxyacetone phosphate, FBP: fructose 1,6 bisphosphate, F6P: fructose-6-phosphate, GAP: glyceraldehyde-3-phosphate, GLU: glutamate, G1P: glucose-1-phosphate, G6P: glucose-6-phosphate, ISO-CIT: isocitric acid, MAL: malic acid, OAA: oxaloacetic acid, PEP: phosphoenolpyruvate, PYR: pyruvic acid, RuBP: ribulose 1,5 bisphosphate, Ru5P: ribulose-5-phosphate, R5P: ribulose-5-phosphate, SBP: sedoheptulose 1,7 bisphosphate, SSA: succinyl semialdehyde, SUC: sucrose, SUCC: succinate, SUC-6-P: sucrose-6-phosphate, S7P: sedoheptulose-7-bisphosphate, UDPG: UDP-glucose, 2PGA: 2-phosphoglyceric acid, and 3PGA: 3-phosphoglyceric acid.

We measured the relative metabolite pools of PCC 11801 and PCC 11802 by isotopic ratio method^29,55,56^. We utilized the intracellular metabolite extract of PCC 11801, which is fully labeled with isotopic ^13^C as an internal standard. This strategy allowed us to use ^13^C isotopologue of each metabolite as its respective internal reference. Although the levels of individual metabolites varied between PCC 11801 and PCC 11802 (Fig. S36), this data alone may not suffice for the identification of flux control points. The individual carbon flux control points might differ in various strains. Therefore, to understand the distinct metabolic changes and differential flux control that occur in PCC 11802 and PCC 11801, resulting in their different growth and biochemical phenotypes, we assessed the fold changes in metabolite levels while shifting from ambient to 1% CO_2_ conditions. A proportionate fold change in the levels of metabolites of a particular pathway is expected with the change in the external conditions (e.g. CO_2_), the absence of which will indicate the presence of a regulatory node or a rate-limiting reaction. The principal component analysis (PCA) performed using the fold change values showed PCC 11802 and 11801 as distinct groups (Fig. S37). In general, we observed that the abundance of metabolites involved in the Calvin-Benson-Bassham (CBB) cycle and participating in CO_2_ fixation was elevated at 1% CO_2_ in both the strains (Fig. 5). However, the enhancement of metabolite levels was greater for PCC 11802 compared to PCC 11801. Despite an increase in abundance of sedoheptulose 1,7 bisphosphate (SBP) in PCC 11801 at 1% CO_2_, the levels of sedoheptulose-7-phosphate (S7P) showed the negligible change between ambient and 1% CO_2_. This indicates that the conversion of SBP to S7P catalyzed by the enzymes sedoheptulose 1, 7 bisphosphatase (SBPase) might be rate-limiting under 1% CO_2._ This might be a reason why increasing the CO_2_ concentration does not result in a similar enhancement of growth rate in PCC 11801 compared to PCC 11802 (Fig. 2A). In fact, cyanobacterial fructose-1,6 -/sedoheptulose-1,7-bisphosphatase (FBP/SBPase) has been identified as one of the carbon flux control enzymes, and its overexpression has been shown to increase the growth rate and biomass accumulation in PCC 6803^59,60^. PCC 11802, on the other hand, has a higher fold increase of both SBP as well as S7P suggesting efficient carbon assimilation and regeneration through the the CBB cycle (Fig. 5).

The higher fold increase of ribulose 1,5 bisphosphate (RuBP) and 3-phosphoglycerate (3PGA) in 11801 may be indicative of slower conversions to downstream metabolites as seen by the comparative lower fold increase of fructose 1,6 bisphosphate (FBP) and fructose-6-phosphate (F6P). This might be indicative of probable triose phosphate utilization (TPU) limitation^11^ in 11801, which might be negligible in PCC 11802. TPU limitation arises when the inherent metabolism of the organism is insufficient to completely utilize the triose phosphates generated from the CBB cycle, or the rate of utilization of triose phosphates is less than the production rate^11^. The extent of TPU limitation may vary in different cyanobacterial strains, and it may be hypothesized that TPU utilization will be more evident under elevated CO_2_ conditions^61^. Although there are no direct reports of overcoming TPU limitation in cyanobacteria, a few reports suggest that the photosynthetic efficiency of recombinant strains of cyanobacteria producing sucrose^62^, 2,3 butanediol^5^, isobutanol^63^, and ethylene^64^ was found to be increased compared to the native wild type strains. The decline in the substrates for carboxylation reactions like RuBP and phosphoenolpyruvate (PEP) in PCC 11802 might be indicative of faster conversion to products, 3PGA, and aspartate (ASP), respectively (Fig. 5).

The spontaneity of a particular reaction depends on the Gibbs’ free energy change and the concentrations of reactants and the products. Appropriate thermodynamic evaluation coupled with the overexpression of the enzymes that replenish the rate-limiting metabolites may increase product titers^65^. For enhanced production of biofuels using cyanobacterial hosts, strains capable of better growth under elevated CO_2_ are preferred so that a greater carbon flux can be rerouted towards the desired products. The hypothesis that PCC 11802 utilizes more carbon for growth rather than for storage compared to PCC 11801 at elevated CO_2_ is further strengthened by monitoring the fold increase of another storage molecule, sucrose. We observe that apart from making more glycogen, PCC 11801 also makes more sucrose as shown by higher levels of UDP-glucose (UDPG), sucrose-6-phosphate (SUC-6-P) and sucrose (SUC) at 1% CO_2_. We also observed a higher fold increase of succinate and glutamate in PCC 11801. The higher fold increase of the rate-limiting the CBB cycle metabolites and less TPU limitation in PCC 11802 coupled with its higher growth rate at 1%, CO_2_ makes it an interesting candidate for heterologous expression and production of biofuels/biochemical.

### Genetic modification of PCC 11802

**Figure 6:**
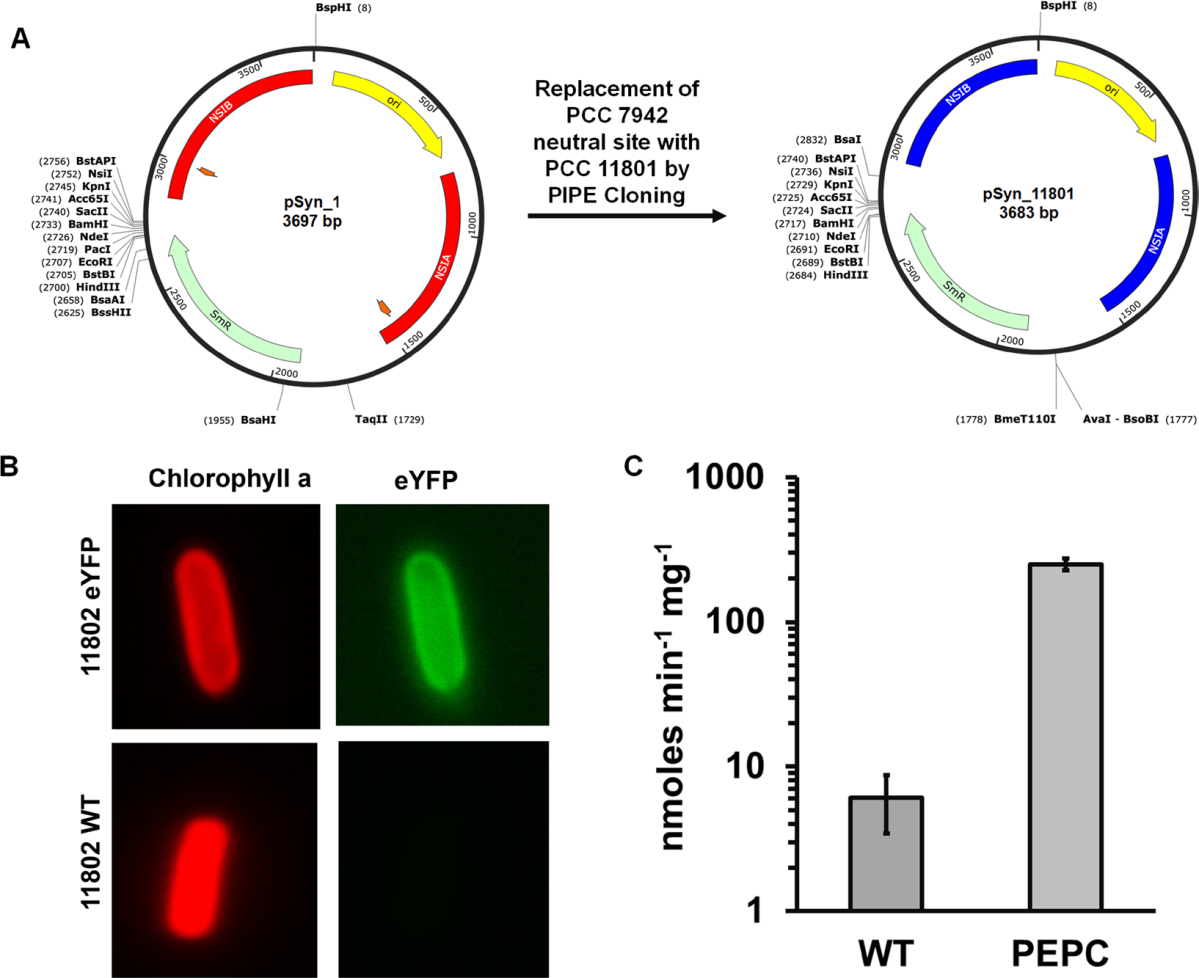
Genetic Modification of PCC 11802. (**A**) Construction of modified vector by replacing the neutral site I (NSI) homology arms of PCC 7942 in the pSyn_1 plasmid with those of PCC 11801 by PIPE cloning method. (**B**) Microscopic images of wildtype and recombinant cells of PCC 11802 showing chlorophyll a fluorescence (left panels) and eYFP fluorescence (right panels), and (**C**) The activity of phosphoenolpyruvate carboxylase (PEPC) protein in wildtype and recombinant cells overexpressing PEPC protein grown under 1% CO_2_ in shake flasks. The activity data is plotted on log_10_ scale.

The ability to carry out genetic modifications is an important pre-requisite for biotechnological applications. Cyanobacterial genomes can be readily engineered via homologous recombination and several vectors are readily available for model cyanobacteria. We first tried the integrative vector pSyn_1 that has been widely used for integration into the neutral site I (NSI) of PCC 7942. This was based on the presence of a putative NSI in PCC 11802 that shares 82% homology with that of PCC 7942. However, the transformation efficiency was too low to be used on a regular basis. To that end, we replaced the homology arms of pSyn_1 with those for NSI of PCC 11801 using polymerase incomplete primer extension (PIPE) cloning^66^ method to obtain the plasmid pSyn _11801 (Fig. 6A). Note that the neutral site regions of PCC 11801, NS1A and NS1B share 100% and 95% identity with the respective regions of PCC 11802. As expected, the modified plasmid shows satisfactory rates of natural transformation in both PCC 11801 and 11802. The transformation rate was found to be 54 ± 10 cfu/µg of the plasmid (eYFP) using 7 mL culture of 0.6 OD_730_.

For high-throughput characterization of biological parts, reporter systems such as enhanced yellow fluorescent protein (eYFP), mOrange, luciferase are commonly used^11,67^. However, metabolic engineering necessitates the expression of heterologous functional genes (enzymes) for the production of value-added chemicals. To enhance the flux towards the desired product, engineering approaches like knockout of storage molecules and overexpression of enzymes crucial for the replenishment of rate-limiting metabolites are essential. Phosphoenolpyruvate carboxylase (PEPC) is a crucial enzyme for an anaplerotic pathway that replenishes the TCA cycle intermediates and is usually overexpressed for enhanced production of biochemical derived from TCA cycle^8,27^. Therefore, we demonstrate the heterologous expression of a reporter and a functional gene encoding for eYFP and PEPC in PCC 11802 under cpcB promoter of PCC 11801 (Fig. 6B,C respectively). These results also show the portability of integrative plasmid and the cpcB promoter of PCC 11801 in PCC 11802.

## Conclusion

We report the physiological, genomic, biochemical, and metabolic characterization of a novel fast-growing and naturally transformable cyanobacterium, *Synechococcus elongatus* PCC 11802. The strain shows a doubling time of 2.8 h under the optimal growth conditions of 1% CO_2_, 38 °C and 1000 µmole photons.m^−2^.s^−1^ and without the addition of any vitamin supplement. PCC 11802 is phylogenetically close to PCC 11801 with ~97% genome identity, and ~97% average protein identity. Both these strains were isolated from the same geographical location (Powai lake, Mumbai, India). Function-based genome comparison shows that these strains differ majorly in toxin-antitoxin systems that are responsible for programmed cell death or reversible growth arrest under unfavorable conditions.

The physiological and biochemical characterization of PCC 11802 showed striking differences compared to PCC 11801. Our results show that the exponential growth of PCC 11802 is better than PCC 11801 under low light-low CO_2_, low light-high CO_2,_ and high light-high CO_2_ conditions. PCC 11802 accumulates ~3 fold more and ~5 fold less glycogen than PCC 11801 under ambient and 1% CO_2_, respectively. PCC 11802 thus diverts more carbon for growth at 1% CO_2_ compared to PCC 11801. We performed metabolomics studies to assess possible reasons for the higher growth rate of PCC 11802 compared to PCC 11801 under high CO_2_ conditions. The results show that PCC 11802 has greater abundance of the CBB cycle intermediates and lesser triose phosphate utilization limitation under elevated CO_2_ condition. The metabolic bottlenecks like repression of SBPase and RuBisCO that are evident in PCC 11801 seem to be alleviated in PCC 11802. Moreover, the reactions involving CO_2_ assimilation catalyzed by RuBisCO and PEPC appear to be more active in PCC 11802.

In terms of metabolic engineering applications, both PCC 11802 and PCC 11801 appear to be promising candidates owing to their faster growth and genetic amenability. However, the choice of strain will depend on the product of interest. We believe that PCC 11802 may be a good candidate for the products that primarily derived from the intermediates of the CBB cycle. PCC 11801, on the other hand, might be a good candidate for the products derived from the TCA cycle. However, a detailed fluxomics study along with a head-on-comparison of product titers in each strain will be required to verify this hypothesis. The unique insights gained from the studies presented in this report strengthen the fundamental understanding of cyanobacterial carbon partitioning and metabolism under carbon limited and sufficient conditions. Furthermore, our study draws attention to the fact that metabolic differences can exist between two very closely related fast-growing cyanobacteria. Also, the metabolic characterization of potential host cyanobacterial strains is necessary to design strain-specific rational engineering efforts rather than a generalized approach.

## Methods

### Isolation, Genome Sequencing, and Genome Annotation of *Synechococcus elongatus* PCC 11802

*Synechococcus elongatus* PCC 11802 was isolated along with PCC 11801 and six other cyanobacterial strains from Powai Lake, Mumbai, India (19.1273°N, 72.9048°E) as described previously^19^. The genomic DNA of PCC 11802 was isolated using a protocol described earlier^19^ and treated with RNAase for removal of RNA contamination before sequencing. The genome sequencing of PCC 11802 was outsourced to Life Technologies (Thermo Fischer Scientific, Waltham, MA, USA) and was sequenced using Ion Torrent Personal Genome Machine (PGM). Other quality checks for raw reads and assembly were as described previously^19^. PCR was performed for filling additional gaps in the genome by designing primers specifically to amplify the gap regions. The whole-genome sequence of PCC 11802 was submitted to GenBank, NCBI (National Center for Biotechnology Information) under accession number, CP034671. The assembled genome was annotated using Integrated Microbial Genomes and Microbiomes, JGI (IMG)^38^, Rapid Annotation using Subsystem Technology (RAST)^35–37^, and NCBI Prokaryotic Genome Annotation Pipeline^39^.

### Phylogenetic analysis

The cDNA of the 16S rRNA sequence was amplified using specific PCR primers. The 16S rRNA sequence of PCC 11802 was submitted to NCBI under accession number, MH666134. The phylogenetic analysis was performed using 16S rRNA and protein sequences of a set of 29 housekeeping genes from PCC 11802 and other cyanobacterial strains as described previously^32,33^. Concatenated sequences of these 29 proteins for 130 strains were obtained from the *CyanoGEBA*^32^ resource. The respective sequences were aligned using Clustal Omega^68^, and the phylogenetic tree was constructed using MEGA7^69^ and iTOL (Interactive Tree Of Life)^70^.

### Comparative genome analysis

The function-based genome comparison was performed using RAST^35–37^ to identify the proteins unique to PCC 11802 compared to PCC 11801. The sequence-based comparison was performed using RAST^35–37^ by using PCC 11802 as a reference against its closest neighbor strains PCC 11801, PCC 7942, UTEX 2973, and distantly related model strains PCC 7002 and PCC 6803. The percent identity between each protein of PCC 11802 and the proteins of the abovementioned strains was calculated. The proteins that gave bi-directional best hits were identified and represented in this study.

The whole-genome of PCC 11802 was used as the reference and aligned against PCC 11801 and PCC 7942 using Mauve tool^71^, and single nucleotide polymorphisms (SNPs) in PCC 11802 were calculated in comparison to PCC 11801 and PCC 7942. The differences between sequences of house-keeping proteins of PCC 11802 to that of respective protein sequences in neighbor strains PCC 11801 and PCC 7942 were identified by multiple sequence alignment using Clustal Omega^68^.

### Transmission electron microscopy (TEM)

One mL of an exponentially growing culture of PCC 11802 and PCC 11801 was centrifuged at 8000 g for 5 min, and the pellet was washed with MiliQ to remove traces of BG-11 medium components. The pellet was re-suspended in MilliQ water, and approx 10 µL of the re-suspended culture was spotted on copper-coated formvar grids (Electron Microscopy Sciences, Hatfield, PA, USA), washed with Milli Q water and air-dried for 20 minutes. These grids were imaged using an EM (Philips CM-200, Amsterdam, Netherlands) at 200 kV with a magnification of Å~ 6600. Images were recorded digitally by using the Keen View Soft imaging system (Olympus, Tokyo, Japan).

### Growth conditions

The strain was maintained in a shaker at a temperature of 38 °C, 120 rpm, ambient (0.04%) CO_2,_ and light intensity of 400 µmole photons.m^−2^.s^−1^ in BG-11 medium (pH = 7.5) unless specified otherwise. The CO_2_ tolerance studies were performed in CO_2_ incubator shaker (Adolf Kuhner AG, LT-X, Birsfelden, Switzerland) at CO_2_ concentrations of 0.04%, 0.5%, 1%, 5%, 10% and 15% in the chamber at 38 °C and 120 rpm. The growth at different CO_2_ concentrations was measured as OD_720_ using one mL of cells in a UV-visible spectrophotometer (Shimadzu, UV-2600, Singapore).

The growth of PCC 11802 under a range of conditions including light intensity, temperature, and CO_2_ concentration was measured in terms of OD_720_ using a multi-cultivator (Photon Systems Instruments, MC 1000-OD, Czech Republic) with an inlet gas flow rate of 500 SCCM to bubble all eight tubes of multi-cultivators. The specific growth rate (µ) was estimated during the exponential growth phase from the slope of the semi-logarithmic plot between OD_720_ nm and time. Doubling time was calculated as ln (2)/µ.

### Carbohydrate and glycogen estimation

The total carbohydrates and glycogen content were measured using PCC 11802 cells harvested at 0.6–0.7 OD_720_ nm at different CO_2_ concentrations (0.04%, 0.5%, and 1%), 38 °C, 400 µmole photons.m^−2^.s^−1^ and 120 rpm as described earlier^19^.

### Preparation of ^13^C isotopically labeled biomass of synechococcus elongatus PCC 11801 for use as internal standard

After several trials, a protocol was developed to obtain metabolite extract that shows dominant ^13^C monoisotopic peaks but no ^12^C monoisotopic peaks for all metabolites. Modified BG-11 medium that does not contain any organic carbon source (henceforth referred to as BG11-C) such as sodium carbonate, citric acid, and ferric ammonium citrate was used to prepare fully ^13^C labeled metabolite extracts of PCC 11801. Iron sulfate heptahydrate was used to provide an iron source. The exponentially growing culture of PCC 11801 pre-adapted to BG11-C medium was used for inoculation with an O_D730_ nm of 0.05 with a culture volume of 20 mL in 100 mL Erlenmeyer flask. Lower biomass was used for inoculation to minimize dilution with ^12^C present in the biomass from the inoculum. A stopper was used to prevent the exchange of ^12^CO_2_ from the environment. Initially, ^13^C-labeled sodium bicarbonate (NaH^13^CO_3_, 98 atom % ^13^C from Sigma-Aldrich, St. Louis, MO) was added at a concentration of 2 g/L in the culture. Additional doses of NaH^13^CO_3_ were provided at 18, 19.5, 21, and 22.5 hours after inoculation at a final concentration of 1 g/L. The ^13^C labeled biomass was harvested at 23 h by fast filtration in the presence of light followed by rapid quenching in 80:20 methanol-water (precooled to −80 °C). The ^13^C labeled metabolites were extracted from quenched cells as described earlier^72^ with a minor modification, viz., the use of extraction solvent volume that was twice as large as earlier. The ^13^C labeled metabolite extracts were filtered, and multiple aliquots of equal volume were dispensed in Eppendorf tubes, lyophilized and stored at −80 °C until ready for use. The ^13^C labeling procedure was carried out in New Brunswick Innova 44 R shaker (Eppendorf, Hamburg, Germany) maintained at 38 °C, 120 rpm, and a light intensity of 300 µmole photons.m^−2^.s^−1^.

### Metabolite profiling using liquid chromatography-mass spectrometry (LCMS)

PCC 11802 and 11801 cells were grown under 0.04% and 1% CO_2_ at 38 °C in shake flasks. Exponentially growing cells at an OD_720_ of ~0.6 were filtered rapidly on nylon membrane filters (Whatman, 0.8 µ) in the presence of light. The cells on the membrane filters were quickly transferred to 80:20 methanol-water (precooled to −80 °C) to quench the metabolism. The metabolites were extracted using a protocol as reported previously^73^. The metabolite extract was lyophilized and stored at −80 °C until ready for LCMS analysis. The metabolite extracts were reconstituted in 100 µL 50:50 methanol-water and filtered using nylon syringe filters to remove any particulate matter. Each sample was mixed with fully ^13^C-labeled biomass of PCC 11801 that acted as an internal standard. An equal volume of labeled internal standard was added to all the samples. The data was acquired using an information-dependent acquisition (IDA) method on Triple-TOF 5600+ instrument (SCIEX, Framingham, MA) interfaced with Shimadzu Ultra Performance- Liquid Chromatography (UPLC) system Nexera LC −30 AD (Shimadzu, Singapore). The instrument was operated under negative ion mode to acquire data in the m/z range of 50–1000 Da. The cycle and accumulation times were 1 s and 250 ms, respectively. Six µL sample was analyzed on reverse-phase ion-pairing chromatography using a C18 Synergi 4 μm Hydro-RP LC column 150 × 2 mm (Phenomenex Inc, Torrance, CA). The gradient program and other instrument parameters were as reported earlier^72^. Peak areas corresponding to the ^12^C monoisotopic peak and ^13^C monoisotopic peak for the metabolites of interest were quantified using the proprietary software MultiQuant (SCIEX, Framingham, MA).

The relative quantification of targeted metabolites under 0.04% and 1% CO_2_ conditions was performed by the normalizing area under the peak for ^12^C monoisotopic peak of a particular metabolite by its respective ^13^C monoisotopic peak to obtain area ratios. The fold change in metabolite pools under 1% compared to 0.04% CO_2_ was then calculated (area ratio under 1% CO_2_/area ratio under 0.04% CO_2_). Statistical analysis and principal component analysis (PCA) were performed, and a heat map constructed using MetaboAnalyst 4.0^74,75^ (Figs. S37 and 38).

### Genetic manipulation

Seven mL of 0.6 OD_730_ culture of PCC 11802 was centrifuged at 4,000 g for 5 minutes at room temperature. The pellet was washed and re-suspended in 100 µL BG-11 medium.

 Two µg pSyn_11801 plasmid, containing the gene of interest (eYFP or PEPC), was added to the resuspended cells. The cell-DNA mixture was incubated at 34°C in the dark for 12 h. The mixture was then spread on a 0.22 µm filter membrane placed on a 1% BG-11 agar plate and incubated for 24 h at 38 °C and 150 µE light. Then the filter membrane was transferred to a BG-11 agar plate containing 50 µg/mL of antibiotic, spectinomycin. After 48 h of incubation, colonies appeared on the filter membrane, which was then patched on a plate containing 100 µg/mL of spectinomycin to achieve complete chromosomal segregation. Segregation was checked using confirmation primers, 5′CCAACGCCTATTCCAAGGGCGGC3′, and 5′TGGCAATGTCTCTCTGAGGGGATG3. All the colonies obtained by transforming pSyn_11801 plasmids were also verified using gene-specific primers.

### Fluorescence microscopy

Fluorescence microscopy was performed as described earlier^19^. Briefly, the wild-type and eYFP expressing PCC 11802 cells (OD_720_ of ~1.0) were centrifuged at 8000 g for 3 min and washed with Milli-Q water twice. The cells were then re-suspended in 4% paraformaldehyde for 30 minutes at 4 °C. The fluorescence images of fixed WT and eYFP mutant cells were acquired using a Zeiss Axio Observer Z1 (100X objectives, NA = 1.40; Carl Zeiss MicroImaging Inc., Oberkochen, Germany) equipped with Axiocam camera controlled by Axiovision software [Axio Vision Release 4.8.3 SP1 (06–2012)]. Exposure time for imaging was 300 ms.

### Measurement of phosphoenolpyruvate carboxylase (PEPC) activity

The wildtype and recombinant PCC 11802 cells containing a gene encoding PEPC protein were grown till OD_730_ of 0.6–0.7 at 1% CO_2_ and 200 µE of light intensity in a shaker (Adolf Kuhner AG, LT-X, Birsfelden, Switzerland)_._ One hundred mL of the culture was harvested by centrifugation at 8000 g for 10 minutes. The pellet was then resuspended in 500 µL of lysis buffer containing 50 mM TRIS HCl (pH 8), 10 mM MgCl_2_, 10% (v/v) glycerol, 1 mM EDTA, 1 mM DTT and 1 mg/L of lysozyme. One mM PMSF was added to minimize proteolysis^76^. The cells were lysed using tissue lyser LT (Qiagen, Hilden, Germany) with 0.1 mm glass beads in 30 cycles of 1 min of bead beating with 1 minute of cooling on the ice between two cycles.

The cell debris was separated by centrifugation at 20,000 g for 30 min at 4 °C, and the soluble crude extract collected. The protein concentration in the extract was determined via Bradford assay using a standard curve for bovine serum albumin (BSA). PEPC activity was measured using a coupled assay where PEPC from crude extracts converts phosphoenolpyruvate (PEP) to oxaloacetic acid (OAA), which is then converted to malate. The second step concomitantly converts NADH to NAD+ and is catalyzed by the added malate dehydrogenase, which was overexpressed in *E. coli* and purified. The reaction mixture thus contained 100 mM Tris-HCl (pH 8), 10 mM NaHCO_3_, 5 mM MgCl_2_, 200 µM NADH, 0.13 µg of purified *E. coli* malate dehydrogenase, 2 mM PEP, and appropriate amounts of the crude extract. The reaction was initiated by addition of the crude extract at 30 °C. The decrease in absorbance at 340 nm due to the conversion of NADH to NAD+ was monitored. The PEPC activity was calculated from the absorbance versus time graph and normalized to the total concentration of protein in the crude extract.

## Supplementary information

Supplementary Information.

Supplementary File S-1.

Supplementary File S-2.

Supplementary File S-3.

Supplementary File S-4.

## Data Availability

All data generated or analyzed during this study are included in this article (and its Supplementary Information Files). The complete genome and 16S rRNA gene of *Synechococcus elongatus* PCC 11802 is available at GenBank under accession numbers, CP034671 and MH666134, respectively. The data files for the metabolomics study of PCC 11802 presented in this article are deposited to the Metabolomics Workbench repository (http://www.metabolomicsworkbench.org/), 10.21228/M89M4D.
