# Supplementary material for: A Novel Cyanobacterium Synechococcus elongatus PCC 11802 has Distinct Genomic and Metabolomic Characteristics Compared to its Neighbor PCC 11801
Source: Sci Rep. 2020 Jan 13;10:191. doi: 10.1038/s41598-019-57051-0 (PMC6957532; doi:10.1038/s41598-019-57051-0)
Supplement: Supplementary file 1 — Supplementary Information. [file 41598_2019_57051_MOESM1_ESM.pdf]

Supplementary Information

A Novel Cyanobacterium *Synechococcus elongatus* PCC 11802 has Distinct Genomic and Metabolomic Characteristics Compared to its Neighbor PCC 11801

Damini Jaiswal[1], Annesha Sengupta[1], Shinjinee Sengupta [1,2], Swati Madhu[1], Himadri B. Pakrasi[3,4,] and Pramod P. Wangikar[1,2,5*]

[1]*Department of Chemical Engineering, Indian Institute of Technology Bombay, Powai, Mumbai 400076, India*

[2]*DBT-PAN IIT Centre for Bioenergy, Indian Institute of Technology Bombay, Powai, Mumbai 400076, India*

[3]*Department of Biology, Washington University, St. Louis, MO 63130*

[4]*Department of Energy, Environmental and Chemical Engineering, Washington University, St. Louis, MO 63130*

[5]*Wadhwani Research Centre for Bioengineering, Indian Institute of Technology Bombay, Powai, Mumbai 400076, India*

[*]*corresponding author email: wangikar@iitb.ac.in*

Table of Contents

Figures and Tables	Page no.
Figure S1	S-3
Figure S2	S-4
Figure S3	S-5
Figure S4	S-5
Figure S5	S-5
Figure S6	S-6
Figure S7	S-6
Figure S8	S-6
Figure S9	S-7
Figure S10	S-7
Figure S11	S-7
Figure S12	S-7
Figure S13	S-8
Figure S14	S-8
Figure S15	S-8
Figure S16	S-8
Figure S17	S-8
Figure S18	S-9
Figure S19	S-9
Figure S20	S-9
Figure S21	S-9
Figure S22	S-9
Figure S23	S-9
Figure S24	S-10
Figure S25	S-10
Figure S26	S-10
Figure S27	S-10
Figure S28	S-11
Figure S29	S-11
Figure S30	S-11
Figure S31	S-11
Figure S32	S-11
Figure S33	S-12
Figure S34	S-12
Figure S35	S-12
Figure S36	S-13
Figure S37	S-14
Figure S38	S-14
Table S1	S-15
Table S2	S-15
Supplementary Files	S-15

Figure S1: The phylogenetic tree constructed based on 16S rRNA sequences showing the lineage of *Synechococcus elongatus* PCC 11802. Nodes supported by bootstrap values of >0.7 are indicated.

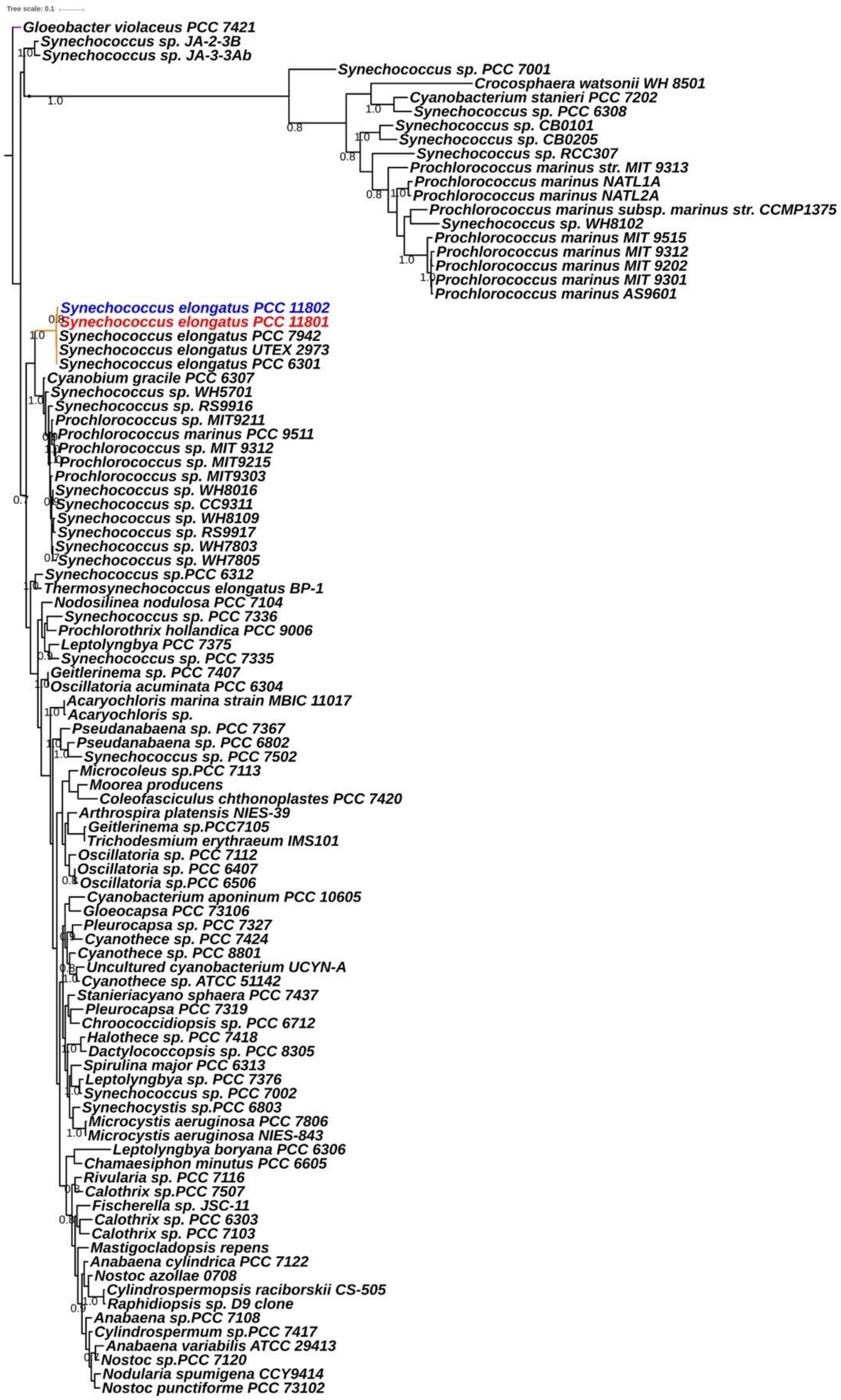

Figure S2: The phylogenetic tree constructed based on concatenated sequences of 29 conserved proteins showing the lineage of *Synechococcus elongatus* PCC 11802. Nodes supported by bootstrap values of >0.7 are indicated.

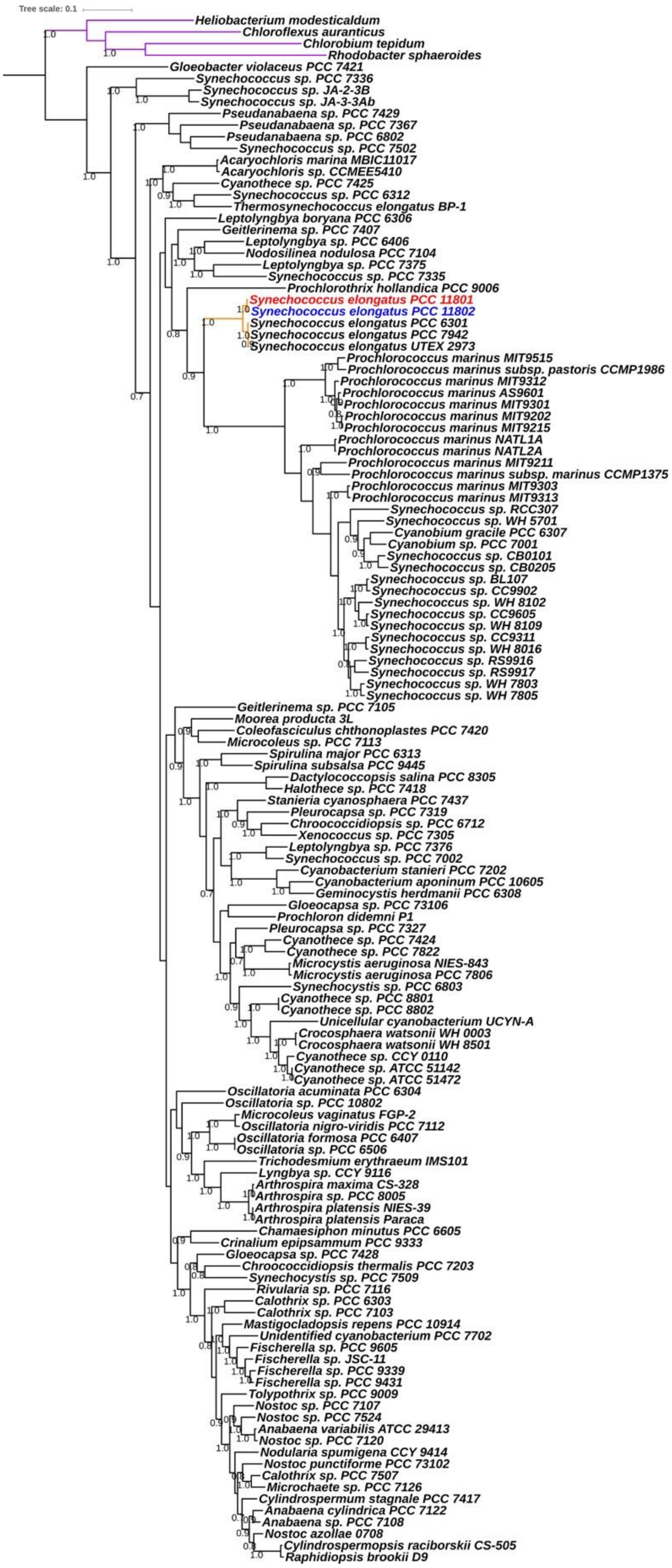

Figure S3: The whole-genome alignment of *Synechococcus elongatus* PCC 11802 with *Synechococcus elongatus* PCC 7942 as reference.

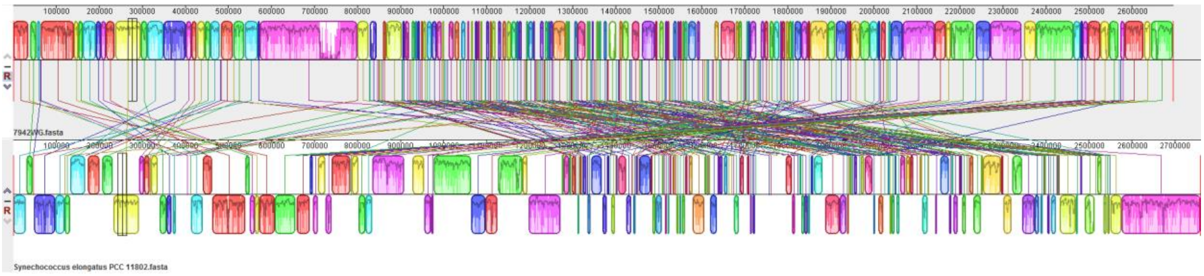

Figure S4: The whole-genome alignment of *Synechococcus elongatus* PCC 11802 with *Synechococcus elongatus* PCC 11801 as reference.

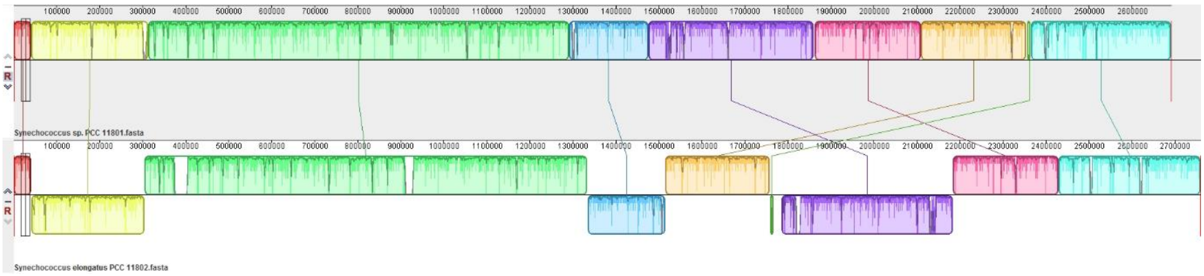

Figure S5: The dot plot between *Synechococcus elongatus* PCC 11802 and *Synechococcus elongatus* PCC 11801

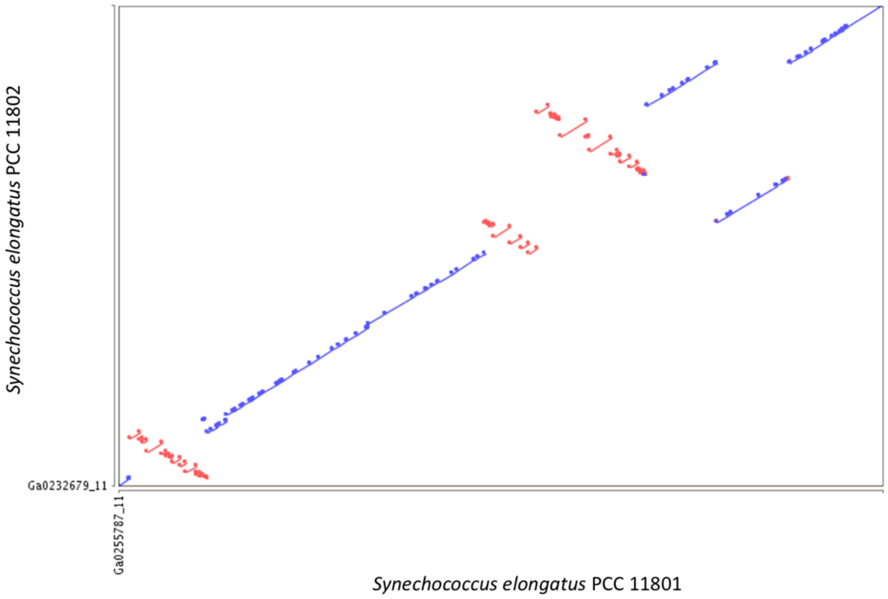

Figure S6: The dot plot between *Synechococcus elongatus* PCC 11802 and *Synechococcus elongatus* PCC 7942

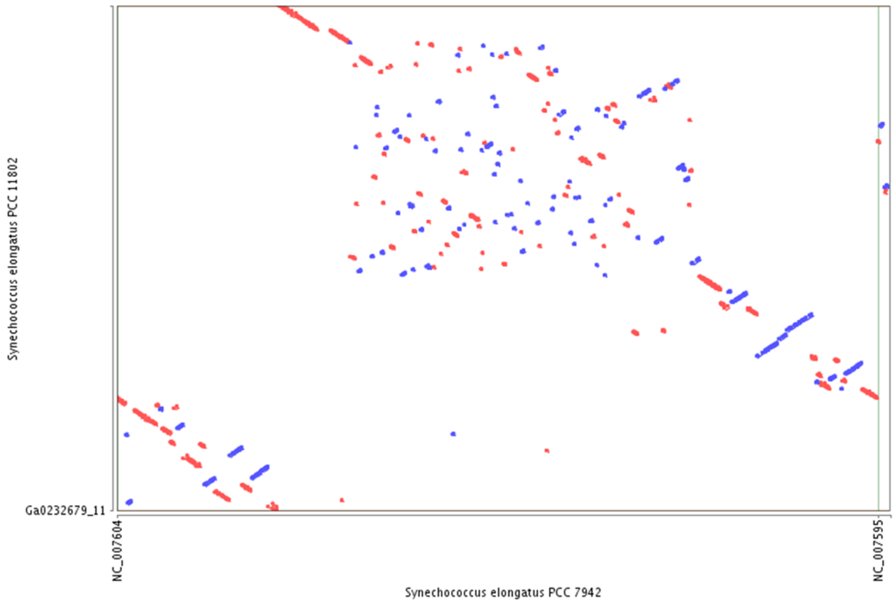

Figure S7: The single amino acid polymorphisms (SAPs) and indels in the sequence of DNA primase (DnaG) protein of PCC 7942 compared to PCC 11802 and PCC 11801

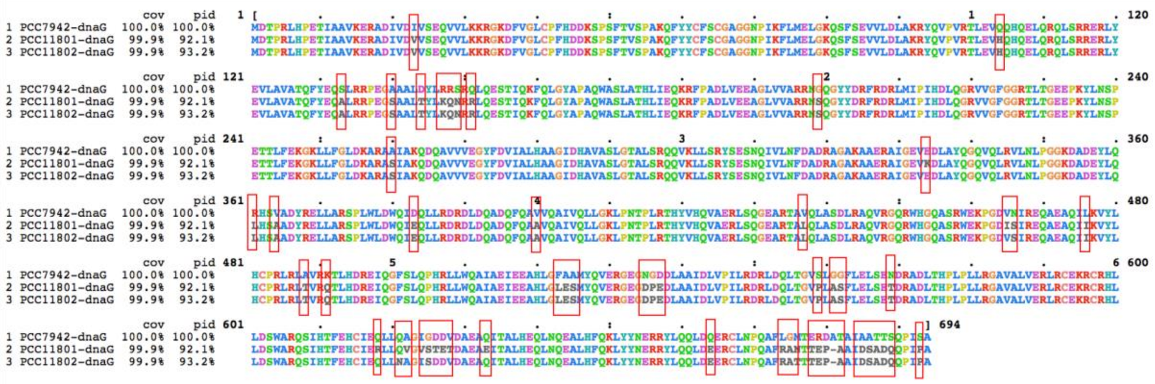

Figure S8: The single amino acid polymorphisms (SAPs) and indels in the sequence of ribosome recycling factor (Frr) protein of PCC 7942 compared to PCC 11802 and PCC 11801

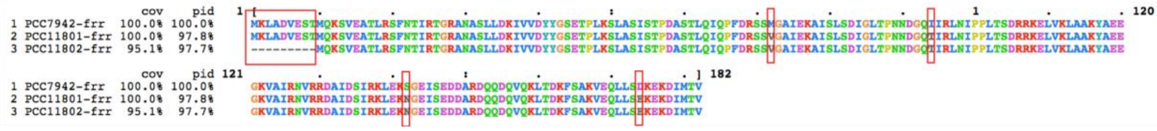

Figure S9: The single amino acid polymorphisms (SAPs) in the sequence of translation initiation factor IF3 (InfC) protein of PCC 7942 compared to PCC 11802 and PCC 11801

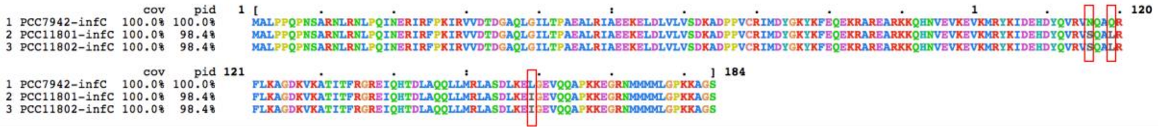

Figure S10: The single amino acid polymorphisms (SAPs) in the sequence of transcription termination factor (NusA) protein of PCC 7942 compared to PCC 11802 and PCC 11801

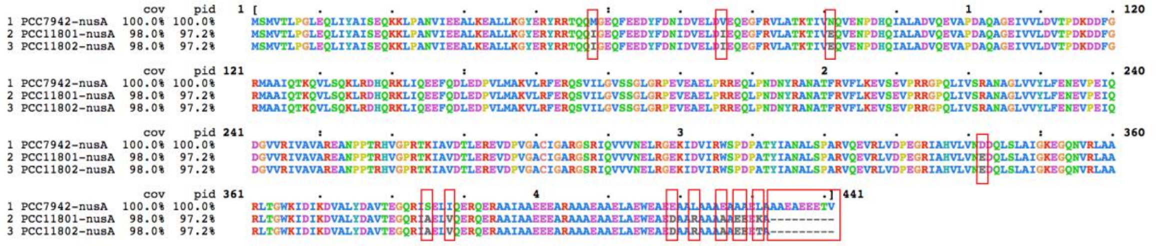

Figure S11: The single amino acid polymorphisms (SAPs) in the sequence of phosphoglycerate kinase (Pgk) protein of PCC 7942 compared to PCC 11802 and PCC 11801

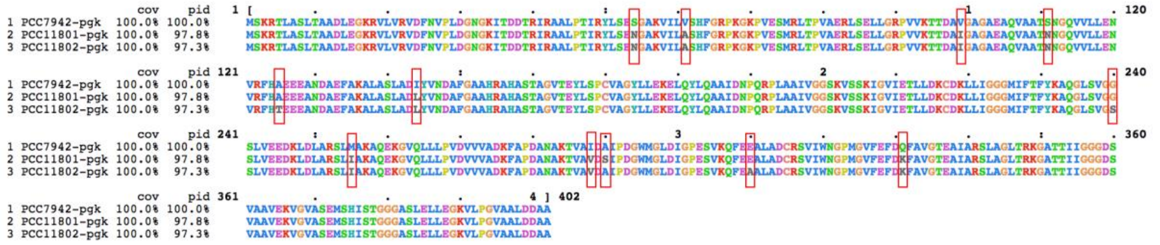

Figure S12: The single amino acid polymorphisms (SAPs) in the sequence of CTP-Synthase (PyrG) protein of PCC 7942 compared to PCC 11802 and PCC 11801

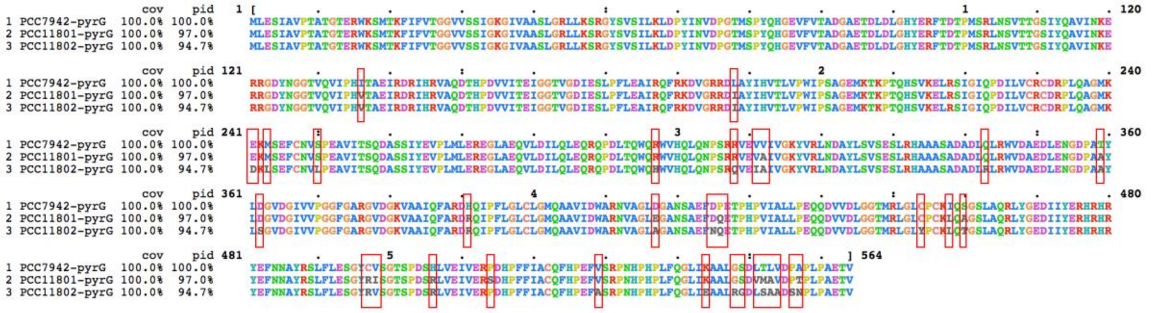

Figure S13: The single amino acid polymorphisms (SAPs) in the sequence of ribosomal protein L1 (RplA) of PCC 7942 compared to PCC 11802 and PCC 11801

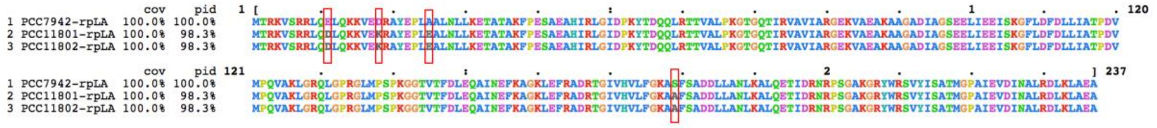

Figure S14: The single amino acid polymorphisms (SAPs) in the sequence of ribosomal protein L2 (RplB) of PCC 7942 compared to PCC 11802 and PCC 11801

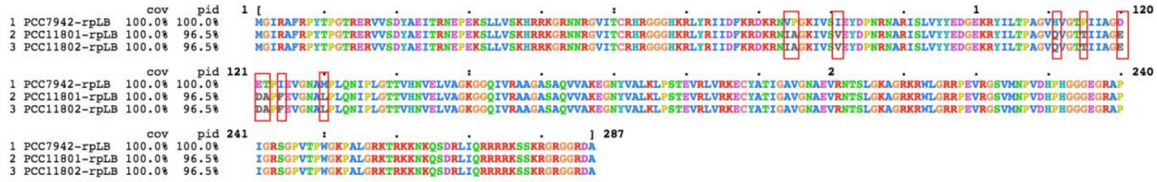

Figure S15: The single amino acid polymorphisms (SAPs) in the sequence of ribosomal protein L3 (RplC) of PCC 7942 compared to PCC 11802 and PCC 11801

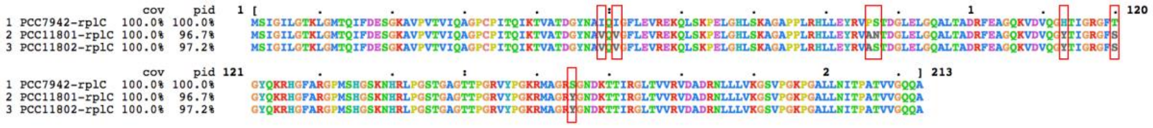

Figure S16: The single amino acid polymorphisms (SAPs) in the sequence of ribosomal protein L4 (RplD) of PCC 7942 compared to PCC 11802 and PCC 11801

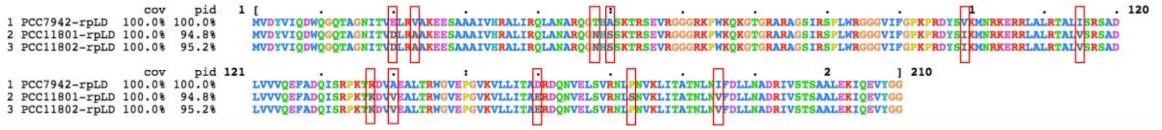

Figure S17: The single amino acid polymorphisms (SAPs) in the sequence of ribosomal protein L5 (RplE) of PCC 7942 compared to PCC 11802 and PCC 11801

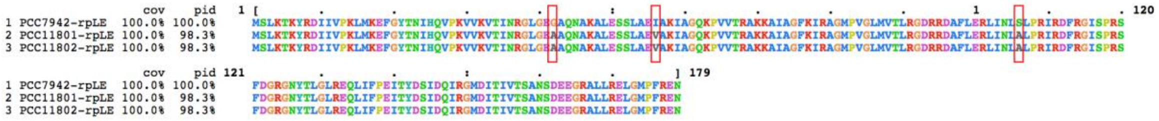

Figure S18: The single amino acid polymorphisms (SAPs) in the sequence of ribosomal protein L6 (RplF) of PCC 7942 compared to PCC 11802 and PCC 11801

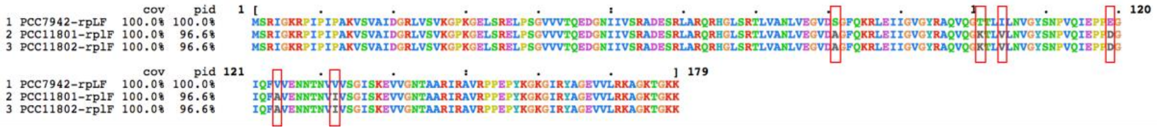

Figure S19: The single amino acid polymorphisms (SAPs) in the sequence of ribosomal protein L7/12 (RplL) of PCC 7942 compared to PCC 11802 and PCC 11801

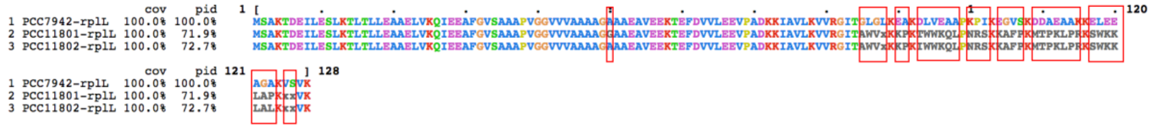

Figure S20: The single amino acid polymorphisms (SAPs) in the sequence of ribosomal protein L13 (RplM) of PCC 7942 compared to PCC 11802 and PCC 11801

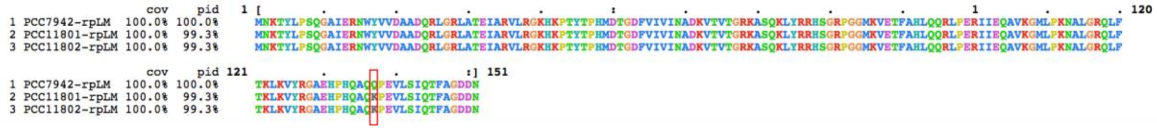

Figure S21: The single amino acid polymorphisms (SAPs) in the sequence of ribosomal protein L13 (RplN) of PCC 7942 compared to PCC 11802 and PCC 11801

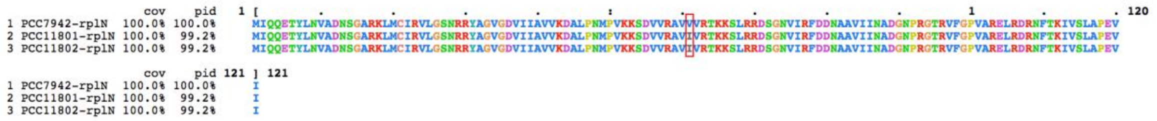

Figure S22: The deletions in the sequences of ribosomal protein L16 (RplP) of PCC 11802 and PCC 11801 compared to PCC 7942

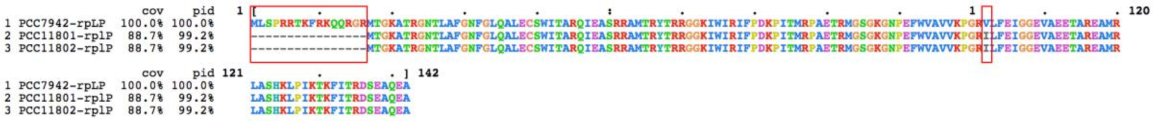

Figure S23: The single amino acid polymorphisms (SAPs) in the sequence of ribosomal protein L19 (RplS) of PCC 7942 compared to PCC 11802 and PCC 11801

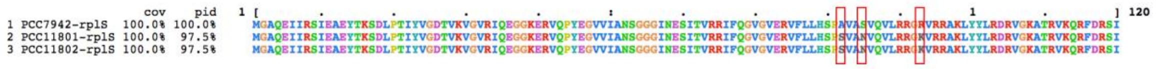

Figure S24: The single amino acid polymorphisms (SAPs) in the sequence of ribosomal protein L20 (RplT) of PCC 7942 compared to PCC 11802 and PCC 11801

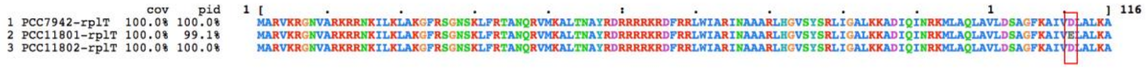

Figure S25: The single amino acid polymorphisms (SAPs) in the sequence of ribosomal protein L27 (RpmA) of PCC 7942 compared to PCC 11802 and PCC 11801

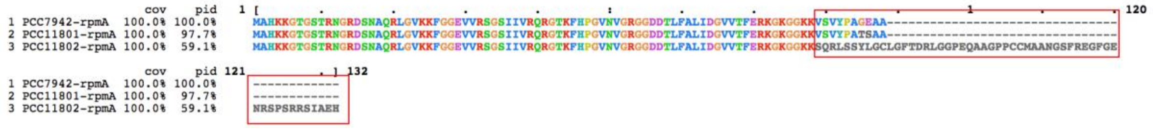

Figure S26: The single amino acid polymorphisms (SAPs) in the sequence of DNA Directed RNA Pol ß subunit (RpoB) of PCC 7942 compared to PCC 11802 and PCC 11801

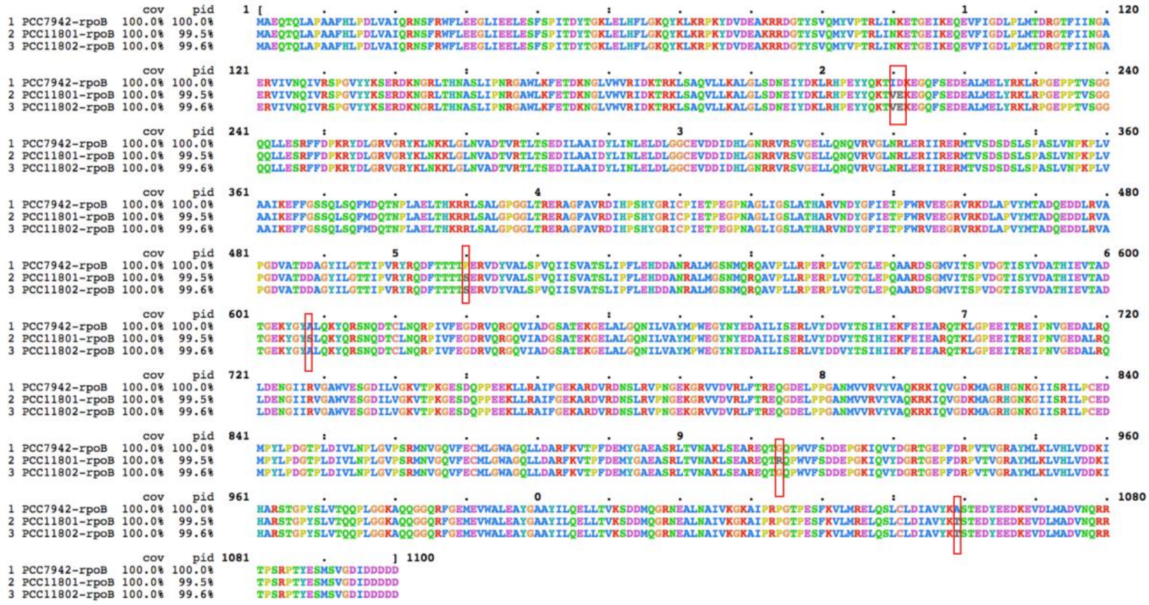

Figure S27: The single amino acid polymorphisms (SAPs) in the sequence of 30S ribosomal protein S2 (RpsB) of PCC 7942 compared to PCC 11802 and PCC 11801

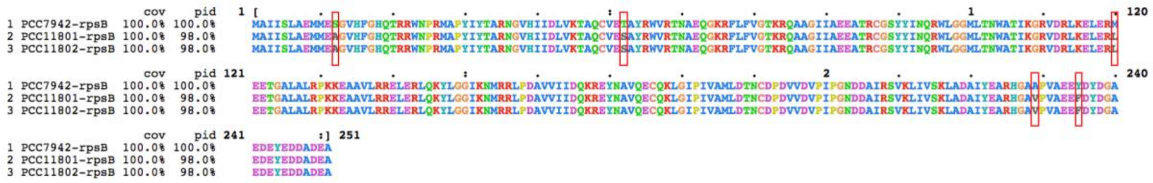

Figure S28: The single amino acid polymorphisms (SAPs) in the sequence of 30S ribosomal protein S3 (RpsC) of PCC 7942 compared to PCC 11802 and PCC 11801

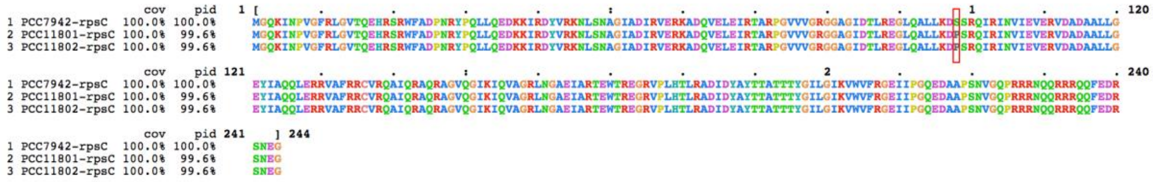

Figure S29: The single amino acid polymorphisms (SAPs) in the sequence of 30S ribosomal protein S5 (RpsE) of PCC 7942 compared to PCC 11802 and PCC 11801

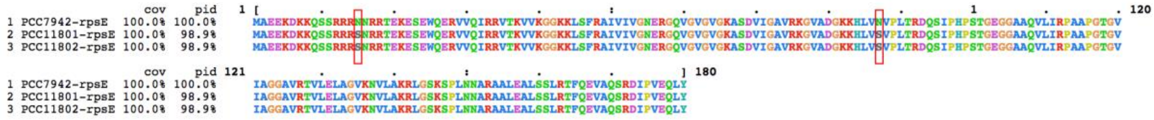

Figure S30: The single amino acid polymorphisms (SAPs) in the sequence of 30S ribosomal protein S9 (RpsI) of PCC 7942 compared to PCC 11802 and PCC 11801

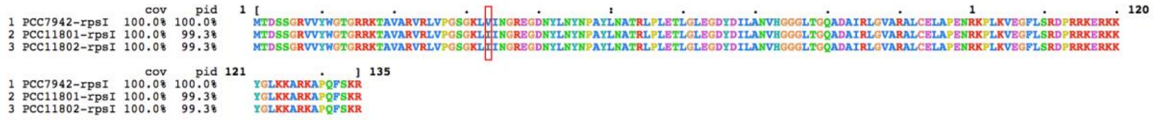

Figure S31: The single amino acid polymorphisms (SAPs) in the sequence of 30S ribosomal protein S11 (RpsK) of PCC 7942 compared to PCC 11802 and PCC 11801

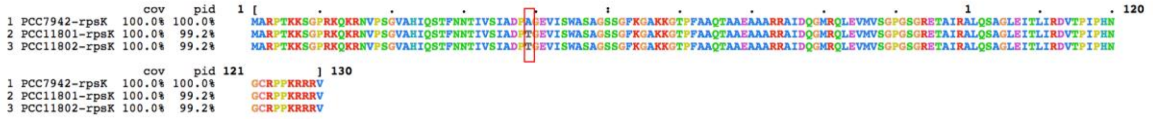

Figure S32: The single amino acid polymorphisms (SAPs) in the sequence of ribosomal protein S13 (RpsM) of PCC 7942 compared to PCC 11802 and PCC 11801

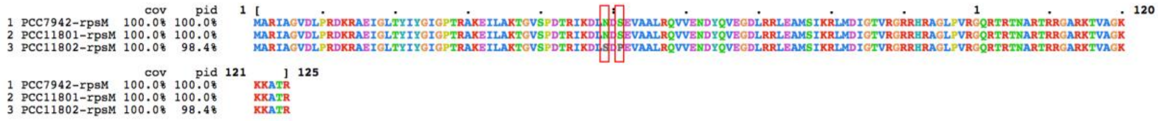

Figure S33: The single amino acid polymorphisms (SAPs) in the sequence of SsrA-binding protein (SmpB) of PCC 7942 compared to PCC 11802 and PCC 11801

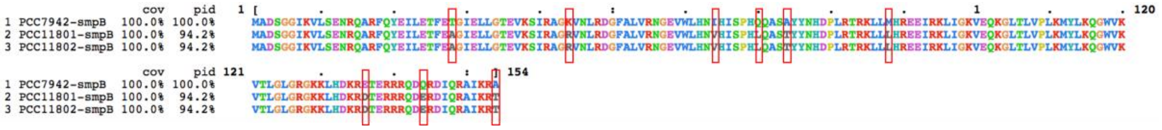

Figure S34: The single amino acid polymorphisms (SAPs) in the sequence of translation elongation factor protein (Tsf) of PCC 7942 compared to PCC 11802 and PCC 11801

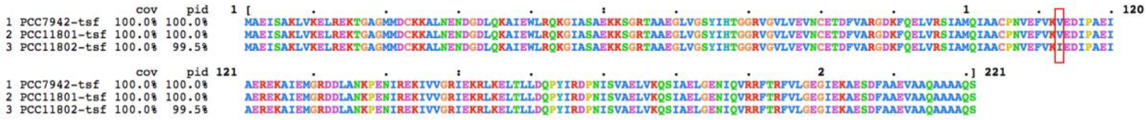

Figure S35: The growth profiles of Synechococcus elongatus PCC 11801 and PCC 11802 at 20°C, 200 µE, and 120 rpm under ambient (0.04%) CO_2 conditions.

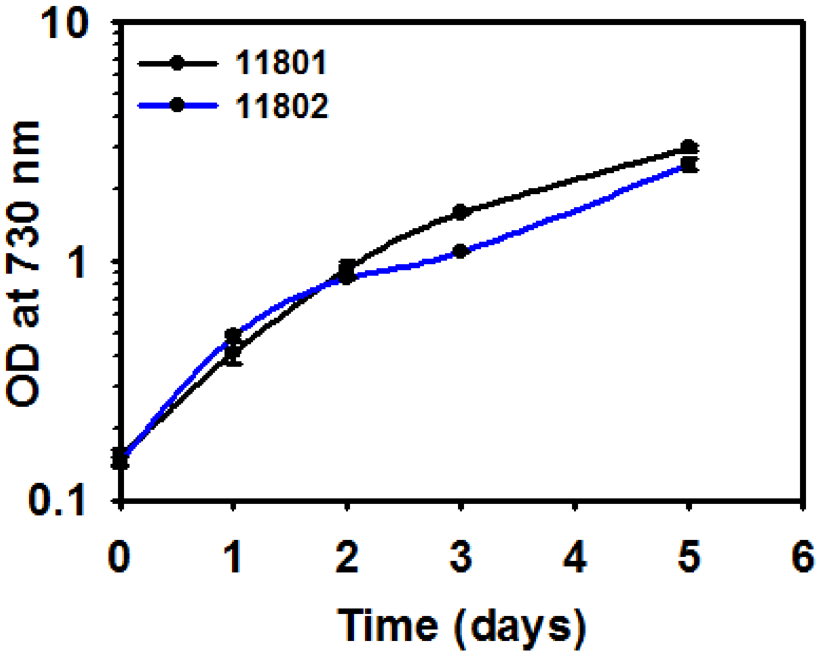

Figure S36: Comparison of relative metabolite pools of PCC 11801 and PCC 11802 under ambient CO_2 conditions. The fully ^{13}C-labeled biomass of PCC 11801 was added as an internal reference in the metabolite extract of both the strains. The area ratios ($^{12}C/^{13}C$) are plotted on Y-axis. Abbreviations used for metabolites, ADPG: ADP-glucose, Ac-CoA : acetyl coenzyme A, ASP : aspartic acid, FBP : fructose 1,6 bisphosphate, F6P : fructose-6-phosphate, GLU: glutamate, G6P : glucose-6-phosphate, PEP : phosphoenolpyruvate, RuBP : ribulose 1,5 bisphosphate, SBP : sedoheptulose 1,7 bisphosphate, SUC: sucrose, SUCC: succinate, SUC-6-P: sucrose-6-phosphate, S7P : sedoheptulose-7-bisphosphate, UDPG: UDP-glucose, and 3PGA : 3-phosphoglyceric acid.

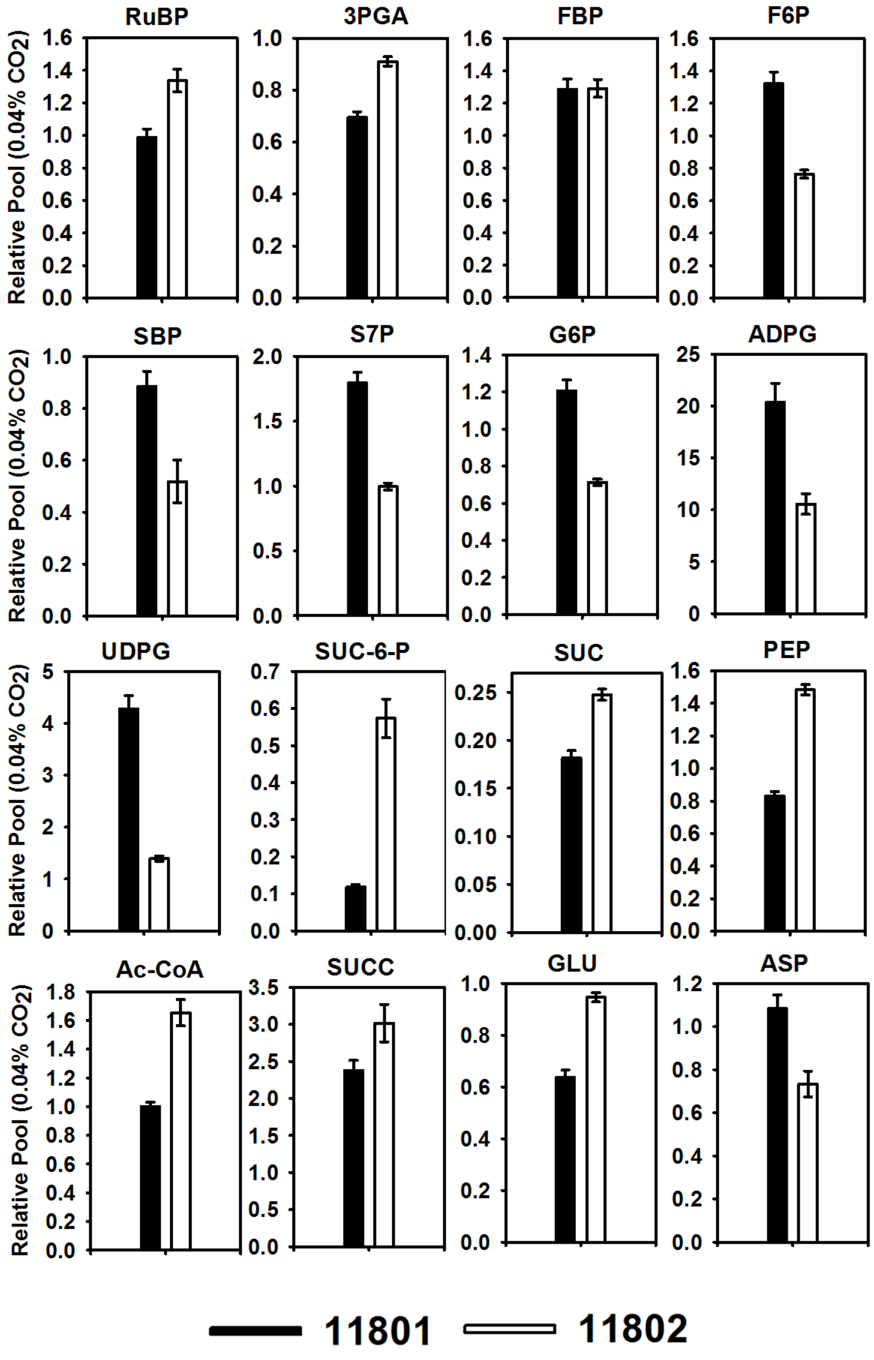

Figure S37: The principal component analysis (PCA) score plot showing PCC 11802 and PCC 11801 as distinct groups based on fold ratio (1 % to 0.04 % CO_2) of metabolites of each group (PCC 11802 and 11801). The area with 95% confidence intervals of each group is highlighted.

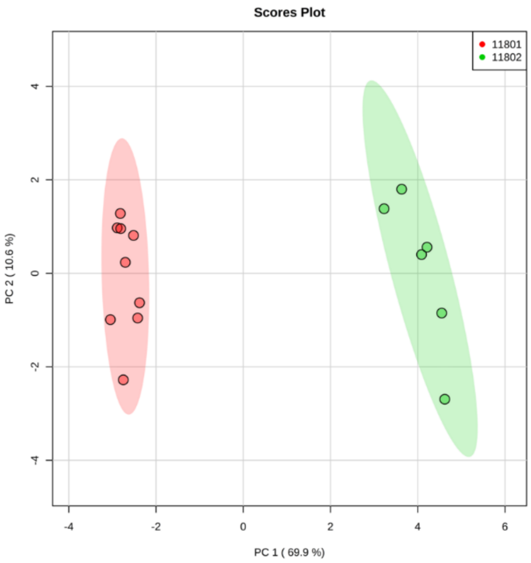

Figure S38: Heat map showing the differential accumulation of metabolites in PCC 11802 compared to PCC 11801 at elevated CO_2 levels (1%). The log_2 fold change of metabolite levels at 1% compared to 0.04% CO_2 was used. Row-wise autoscaling has been performed and the heat map was created using MetaboAnalyst tool.

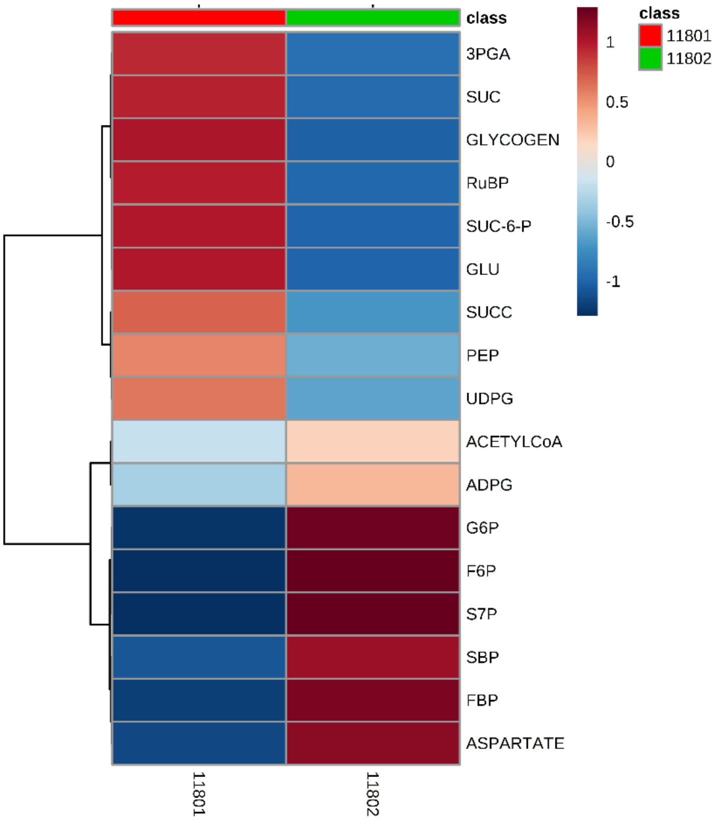

Table S1: The sequence-based comparison between the genome of PCC 11802 with other cyanobacterial strains obtained using RAST tool.

Protein percent identity	Number of homologous genes				
	PCC 11801	PCC 7942	UTEX 2973	PCC 6803	PCC 7002
No hits/ Unidirectional hits	557	465	470	1106	1163
Bidirectional hits					
20-50%	4	8	8	622	573
50-60%	3	13	13	433	416
60-70%	6	42	42	379	397
70-80%	9	177	176	252	251
80-90%	54	686	682	85	77
90-100%	2250	1492	1492	6	6

Table S2: Doubling times of *Synechococcus elongatus* PCC 11802 under different light, temperature, and CO_2 conditions. The highlighted value refers to the least doubling time obtained for PCC 11802.

			Light Intensity (µmole photons.m^{-2}.s^{-1})					
		CO_2 (v/v)	200	300	400	600	800	1000
Temperature	38	0.04%	8.96±1.48	6.74±0.28	6.41±0.43	4.77±0.34	3.58±0.34	3.15±0.27
		1%	9.55±0.40	6.53±0.00	5.79±0.13	4.42±0.17	3.5±0.12	**2.87±0.14**
		3%	-	-	6.1±1.0	4.79±0.10	4.18±0.12	3.7±0.28
	41	1%	-	-	4.83±0.27	3.75±0.32	3.47±0.06	2.98±0.23
		3%	-	-	5.42±0.11	4.65±0.24	4.41±0.35	3.67±0.16
	43	1%	-	-	5.07±0.29	3.75±0.46	3.44±0.26	3.21±0.23
		5%	-	-	-	-	-	3.57±0.92

Supplemental Files

Supplementary File S-1: Complete genome annotation of *Synechococcus elongatus* PCC 11802 obtained from RAST (Table S3), IMG (Table S4) and NCBI Prokaryotic Genome Annotation Pipeline (Table S5).

Supplementary File S-2: Single Nucleotide Polymorphisms (SNPs) in *Synechococcus elongatus* PCC 11802 using *Synechococcus elongatus* PCC 11801 as a reference

Supplementary File S-3: Single Nucleotide Polymorphisms (SNPs) in *Synechococcus elongatus* PCC 11802 using *Synechococcus elongatus* PCC 7942 as a reference

Supplementary File S-4: The sequences of neutral sites (NS1A and NS1B) from *Synechococcus elongatus* PCC 11801
